# Iridium(VII)–Corrole Terminal Carbides Should Exist as Stable Compounds

**DOI:** 10.1021/acsorginorgau.1c00029

**Published:** 2021-12-14

**Authors:** Jeanet Conradie, Abraham B. Alemayehu, Abhik Ghosh

**Affiliations:** † Department of Chemistry, UiT The Arctic University of Norway, N-9037 Tromsø, Norway; ‡ Department of Chemistry, University of the Free State, P.O. Box 339, Bloemfontein 9300, Republic of South Africa

**Keywords:** iridium(VII), terminal carbide, carbide, high-valent, rhenium

## Abstract

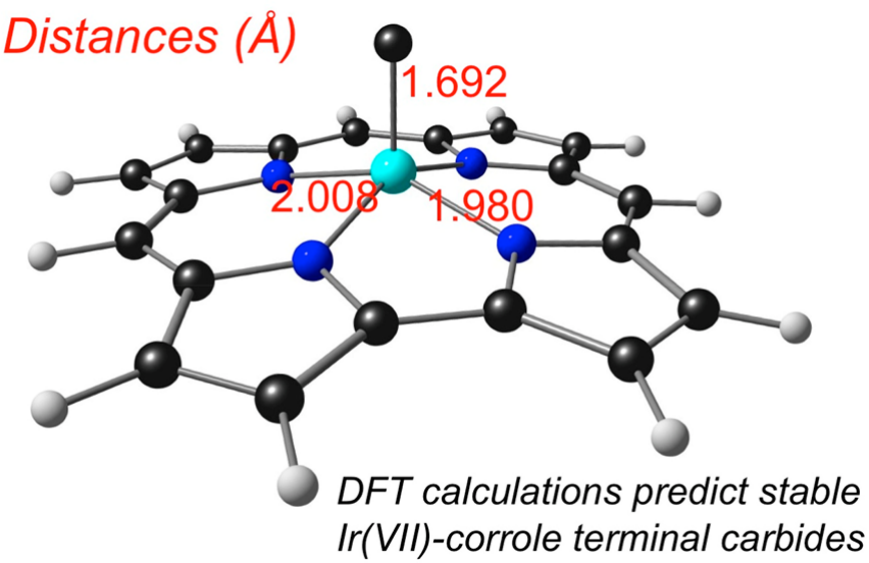

Scalar-relativistic DFT calculations with multiple exchange-correlation functionals and large basis sets foreshadow the existence of stable iridium(VII)–corrole terminal carbide derivatives. For the parent compound Ir[Cor](C), OLYP/STO-TZ2P calculations predict a short Ir–C bond distance of 1.69 Å, a moderately domed macrocycle with no indications of ligand noninnocence, a surprisingly low electron affinity of ∼1.1 eV, and a substantial singlet–triplet gap of ∼1.8 eV. These results, and their essential invariance with respect to the choice of the exchange-correlation functional, lead us to posit that Ir(VII)–corrole terminal carbide complexes should be isolable and indefinitely stable under ambient conditions.

The 5d transition metals have long provided fertile grounds for chemists’ to search for ultrahigh oxidation states, thanks to the strong relativistic destabilization of the 5d orbitals.^1,2^ Notable examples of experimentally verified ultrahigh oxidation states include the Ir^IX^O_4_^+^ cation,^3,4^ Au^V^F_6_^–,^^5,6^ Au^V^_2_F_10_,^7^ and Hg^IV^F_4_.^8^ Quantum chemical studies predict a number of additional as yet experimentally unconfirmed examples of such species,^2^ as well as the 6d complexes Rg^VII^F_7_^9^ and Cn^IV^F_4_.^10^ In a less exotic regime, porphyrin-type ligands have long been known to stabilize high oxidation states such as Fe(IV), Mn(V), Cr(V), Ru(VI), and Os(VI). Recently, corroles have yielded rugged high-oxidation-state complexes, including Ru^VI^N^11^ and Os^VI^N^12,13^ derivatives and square-antiprismatic chiral Mo(VI)^14^ and W(VI)^15,16^ biscorroles. Metal oxidation states above +6, however, have proven elusive among metalloporphyrin-type complexes.^17,18^ Thus, DFT calculations suggest that Re^VII^O_2_ and Ir^VII^O_2_ corrole derivatives are unlikely to exist as stable metal-dioxo species.^19^ In a tantalizing experimental study, a putative Re^VII^[BCP](O)_2_ complex, where BCP^3–^ is a trianionic benzocarbaporphyrin ligand, turned out to be a ligand-oxidized Re^V^[BCPO](O) species instead.^19^ DFT calculations now suggest—with a strong degree of certitude, in our considered opinion—that iridium(VII)–corrole terminal carbides should exist as stable “bottleable” species.^20,21^

Scalar-relativistic DFT calculations^22,23^ (Table 1; see the Supporting Information for details) on Ir[Cor](C) with the OLYP^24,25^ and B3LYP^26,27^ functionals and all-electron ZORA-STO-TZ2P^28,29^ basis sets reveal the structural and electronic hallmarks of a highly stable metallocorrole derivative. The optimized structure (Figure 1), regardless of the functional, exhibits a short Ir–C distance of ∼1.69 Å, which is in excellent accord with that expected on the basis of Pyykkö’s covalent triple-bond radii for Ir (1.07 Å) and C (0.60 Å).^30−32^ The absence of bond length alternations in the corrole skeleton also rule out a noninnocent corrole,^33−37^ indirectly supporting an Ir^VII^C center. The classic Gouterman-type HOMOs,^38−41^ with little metal character, also support the same conclusion (Figure 2). Furthermore, an almost classic corrole-based LUMO suggest a modest electron affinity (EA, Figure 3). Indeed, the calculated adiabatic EA (OLYP) turned out to be only 1.15 eV, essentially the same as (or even lower than) that of a redox-innocent metalloporphyrin such as a nickel or zinc porphyrin.^42,43^ The calculations also indicate a surprisingly large adiabatic singlet–triplet gap of 1.8 eV, another hallmark of a stable closed-shell species (Figure 3). Finally, the near-quantitative agreement between OLYP and B3LYP, two functionals, one pure and the other hybrid, that often yield divergent results,^44−48^ greatly bolster our confidence in the calculated results, in particular our postulate that iridium(VII)–corrole terminal carbides should exist as stable “bottleable” species.^49−51^

**Figure 1:**
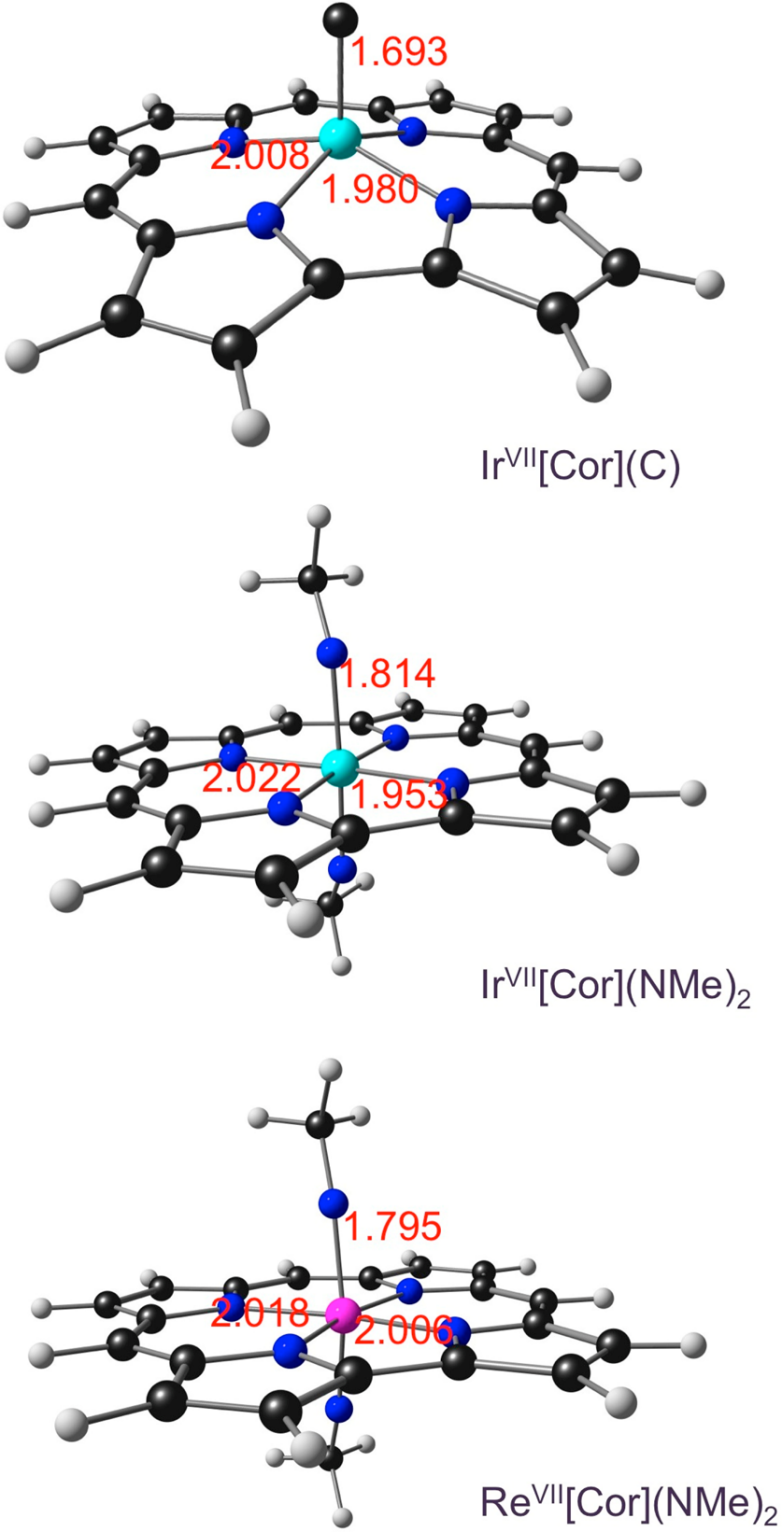
Selected M(VII) porphyrin derivatives with predicted singlet ground states.

**Table 1:** Adiabatic Electron Affinities, Singlet–Triplet Gaps, and HOMO-LUMO Gaps (eV) for Selected M(VII) Corrole Derivatives

	EA		*E*_S–T_		HOMO–LUMO gap	
complex	OLYP	B3LYP	OLYP	B3LYP	OLYP	B3LYP
Ir[Cor](C)	1.16	1.27	1.81	1.78	1.88	2.83
Ir[Cor](NMe)_2_	1.76	1.72	0.86	1.40	0.92	1.90
Re[Cor](C)	2.66	3.11	–0.17	–0.16	0.46	1.50
Re[Cor](NMe)_2_	2.23	2.16	0.54	0.42	0.48	0.21

**Figure 2:**
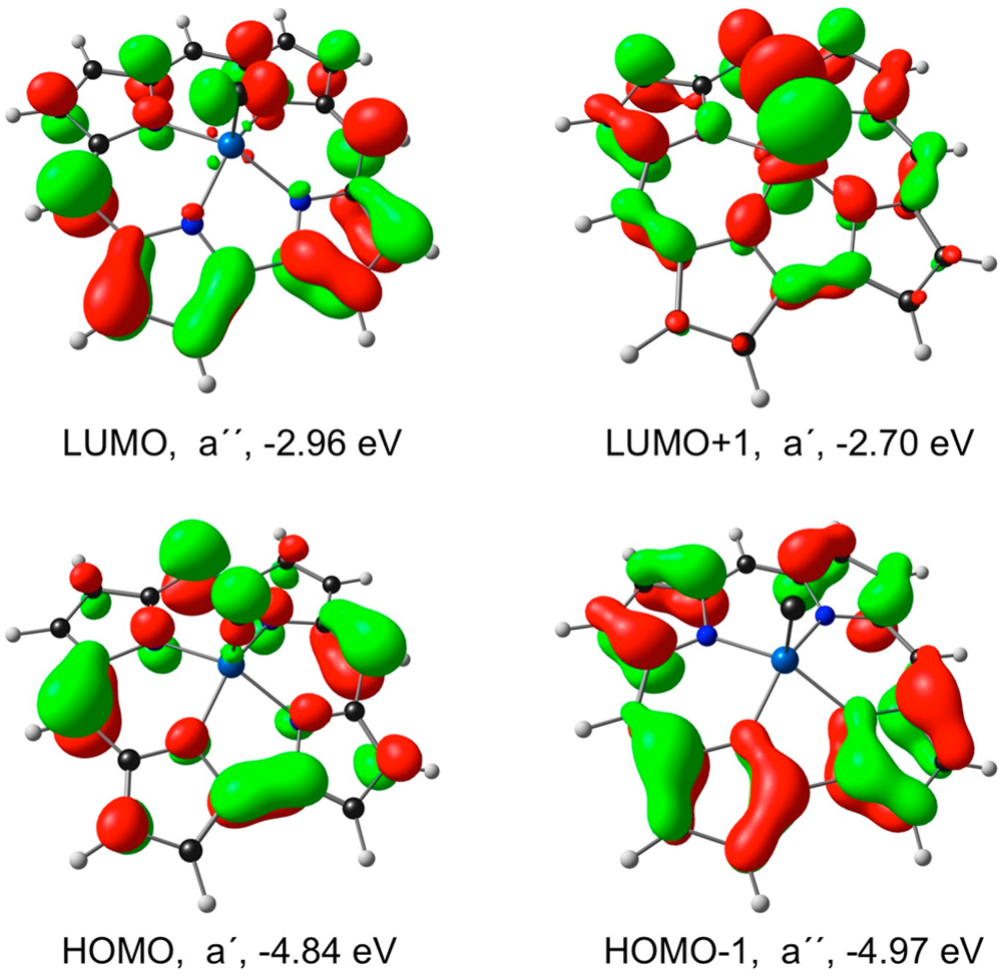
OLYP/STO-TZ2P frontier MOs of Ir[Cor](C), including their *C*_*s*_ irreducible representations and orbital energies.

**Figure 3:**
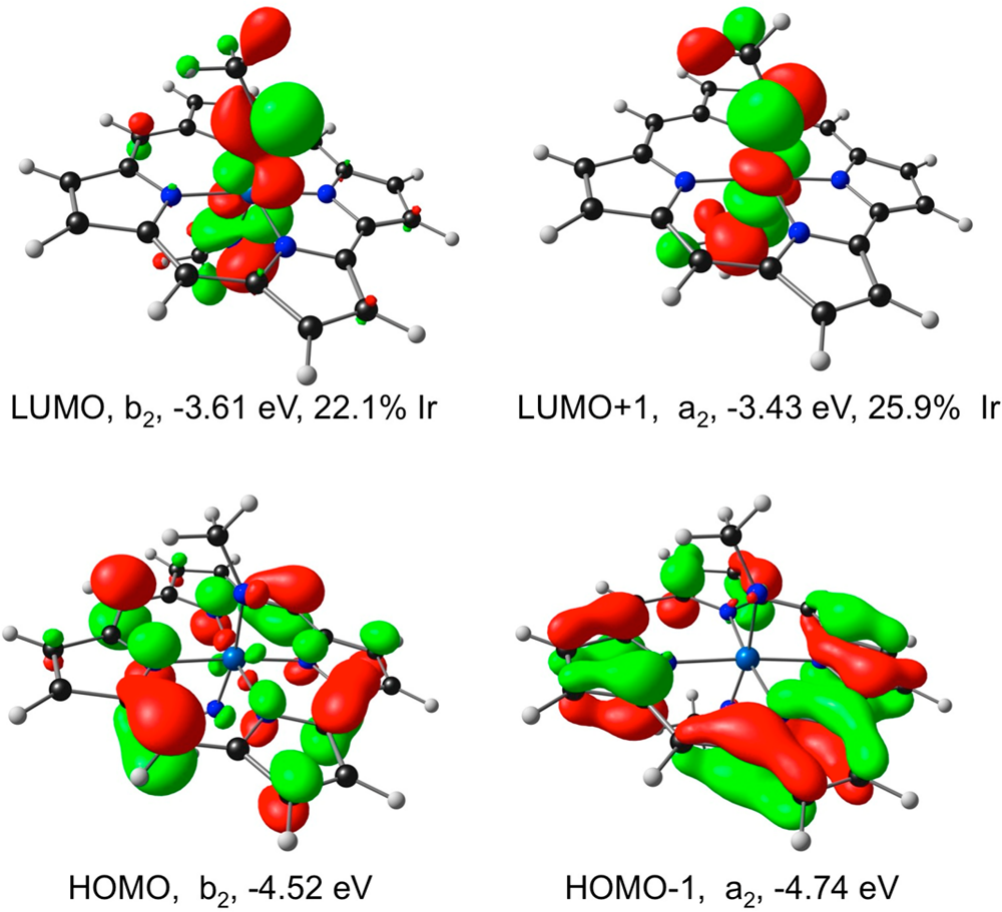
OLYP/STO-TZ2P frontier MOs of Ir[Cor](NMe)_2_, including their *C*_*s*_ irreducible representations and orbital energies.

Compared with Ir[Cor](C), our calculations on Ir[Cor](NMe)_2_ and Re[Cor](NMe)_2_ indicate far higher EAs (1.78 and 2.19 eV, respectively) with OLYP, implying a much greater susceptibility to reduction and nucleophilic attack. Low adiabatic singlet–triplet gaps (0.84 and 0.67 eV, respectively) also foreshadow substantial reactivity. These differences relative to Ir[Cor](C) are primarily ascribable to lower LUMO energy levels and smaller HOMO–LUMO gaps for the two bisimido complexes. Unlike the essentially corrole-based LUMO of Ir[Cor](C), the LUMO Ir[Cor](NMe)_2_ is almost exclusively localized on the Ir(NMe)_2_ axis (Figure 3). The nature and orbital energy of the LUMO, in our view, is the crux of the electronic difference between Ir[Cor](C) and Ir[Cor](NMe)_2_ and key to the predicted high stability of the former.

We further examined the thermodynamic stability of the three M(VII) complexes Ir[Cor](C), Ir[Cor](NMe)_2_, and Re[Cor](C) by calculating the energetics of their reactions with Me_3_P or Me_3_PO, which lead to recognizably stable products (Table 2). Only electronic energies were used for this purpose. It is clear that much smaller and negative Δ*E* values are associated with the reactions of Ir[Cor](C) relative to those of Ir[Cor](NMe)_2_ and Re[Cor](C), clear evidence of the unique thermodynamic stability of Ir[Cor](C).

**Table 2:** Thermochemistry of Some Hypothetical Reactions Involving Ir[Cor](C) and M[Cor](NMe)_2_ (M = Re, Ir)

		Δ*E* (eV)		Δ*E* (kcal/mol)	
number	reaction	OLYP	B3LYP	OLYP	B3LYP
1	Ir[Cor](C) + Me_3_PO → Ir[Cor](PMe_3_) + CO	–1.30	–1.68	–29.9	–38.7
2	Ir[Cor](C) + Me_3_PO → Ir[Cor](CO) + Me_3_P	–1.40	–1.41	–32.2	–32.6
3	Ir[Cor](C) + Me_3_PO → Ir[Cor](CO)(Me_3_P)	–1.72	–2.20	–39.6	–50.7
4	Ir[Cor](NMe)_2_ + PMe_3_ → Ir[Cor](Me_3_P) + *trans*-MeN=NMe	–3.11	–3.93	–71.7	–90.6
5	Re[Cor](NMe)_2_ + PMe_3_ → Re[Cor](NMe) + Me_3_P=NMe	–4.63	–6.08	–106.7	–140.2

In view of the large number of stable Re(VII) complexes known to chemists, it may seem surprising that the prospects for a stable singlet Re[Cor](C) species are almost nonexistent. Thus, both OLYP and B3LYP predict exceptionally high electron affinities >2.5 eV and negative adiabatic singlet–triplet gaps, i.e., triplet ground states (Table 1). Unlike the well-known *fac*-Re^VII^O_3_^+^^52−55^ and [ReH_9_]^2–^^56−58^ architectures, a square-pyramidal geometry with an apical carbide simply does not bring about an adequate HOMO–LUMO gap for a stable singlet ground state; the culprit here is an exceptionally low-energy Re(d_*xy*_)-based LUMO^59^ (which is understandable given the high Re(VII) oxidation state), which translates to the anion and triplet spin density profiles depicted in Figure 4.

**Figure 4:**
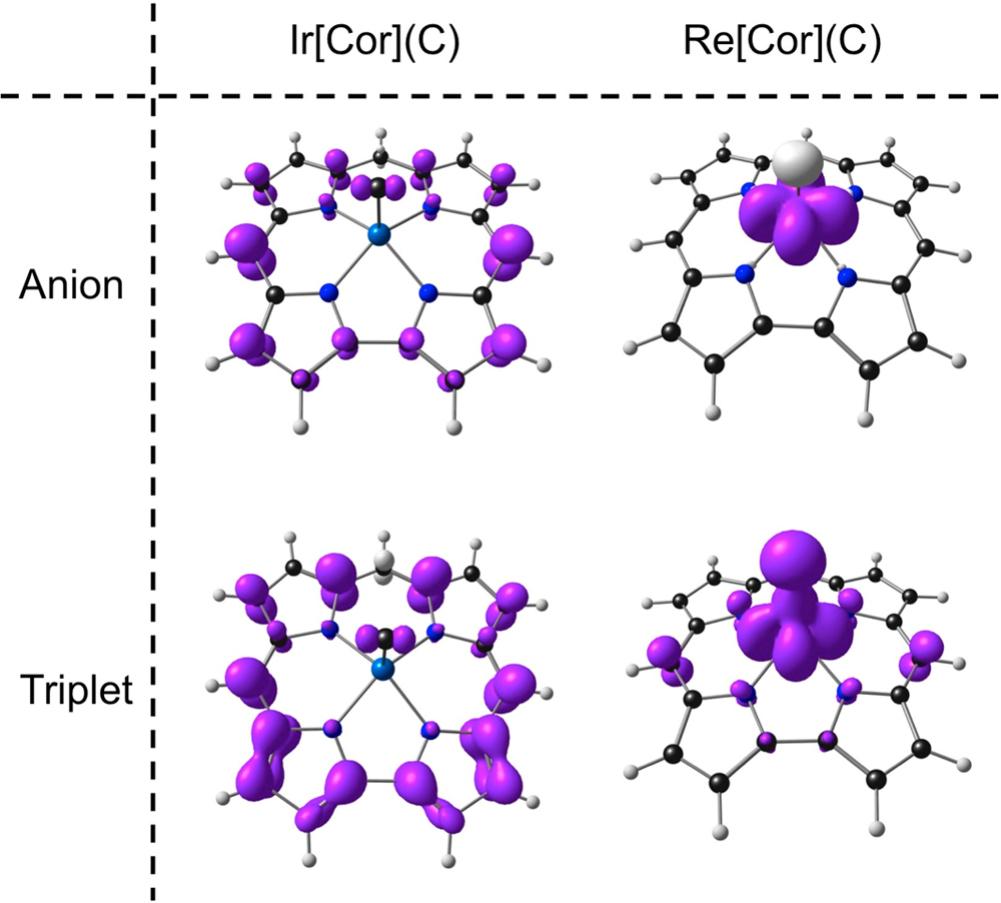
OLYP/STO-TZ2P spin density plots for the anion and triplet states of Ir[Cor](C) and Re[Cor](C). Majority and minority spin densities are depicted in purple and ivory, respectively.

In summary, multiple considerations, including those of electron affinity and singlet–triplet gaps, lead us to postulate the existence of iridium(VII)–corrole carbides as highly stable species, a prediction of interest to not only high-oxidation-state chemistry but also to the field of transition metal carbides.^60^ Terminal carbides are exceptionally rare, with only a handful of examples to date from groups 6 and 8, including [{N(R)Ar}_3_Mo(≡C:)]^−^, [Tp*(CO)_2_M(≡CLi)] (Tp* = 3,5-dimethyltris(pyrazolyl)borate; M = W, Mo),^61−64^ [M(≡C:)(L)_2_(X)_2_] (M = Ru, Os),^65−68^ and [P_2_Mo(≡C:)(CO)]^+,0,–^ (where P_2_ is a terphenyl-diphosphine ligand).^69,70^ The present predictive study, if experimentally realized, would afford the first example of a group 9 terminal carbide.

## Supporting Information Available

Computational methods, sample input, and optimized DFT coordinates (PDF)
