# Supplementary material for: Iridium(VII)–Corrole Terminal Carbides Should Exist as Stable Compounds
Source: ACS Org Inorg Au. 2021 Dec 14;2(2):159–63. doi: 10.1021/acsorginorgau.1c00029 (PMC9955125; doi:10.1021/acsorginorgau.1c00029)
Supplement: Supplementary file 1 — gg1c00029_si_001.pdf [file gg1c00029_si_001.pdf]

Supporting information

Iridium(VII)-Corrole Terminal Carbides Should Exist as Stable Compounds

Jeanet Conradie,[a,b] Abraham B. Alemayehu[a] and Abhik Ghosh*,[a]

[a] Department of Chemistry, UiT – The Arctic University of Norway, N-9037 Tromsø, Norway; Email: abhik.ghosh@uit.no
[b] Department of Chemistry, University of the Free State, P.O. Box 339, Bloemfontein 9300, Republic of South Africa.

Table of Contents

A. Computational method **4**

B. Example input file **5**

C. Optimized all-electron OLYP/ZORA-STO-TZ2P Cartesian coordinates (Å) **7**

1. Ir[Cor](C); C_s; S = 0; Total bonding energy = -248.34690085 eV 7
2. Ir[Cor](C); C_s; S = 1; Total bonding energy = -246.54046220 eV 7
3. {Ir[Cor](C)}$^-$; C_s; S = ½; Total bonding energy = -249.50413800 eV 8
4. Ir[Cor](NMe)$_2$; C_{2v}; S = 0; Total bonding energy = -292.95974054 eV 9
5. Ir[Cor](NMe)$_2$; C_{2v}; S = 1; Total bonding energy = -292.10392868 eV 10
6. {Ir[Cor](NMe)$_2$}$^-$; C_{2v}; S = ½; Total bonding energy = - -294.71565569 eV 11
7. Re[Cor](C); C_s; S = 0; Total bonding energy = -249.53386250 eV 12
8. Re[Cor](C); C_s; S = 1; Total bonding energy = -249.70563716 eV 13
9. {Re[Cor](C)}$^-$; C_s; S = ½; Total bonding energy = - -252.36158143 eV 14
10. Re[Cor](NMe)$_2$; C_{2v}; S = 0; Total bonding energy = -296.38964416 eV 14
11. Re[Cor](NMe)$_2$; C_{2v}; S = 1; Total bonding energy = -295.84740414 eV (non Aufbau solution) 15
12. {Re[Cor](NMe)$_2$}$^-$; C_{2v}; S = 1/2; Total bonding energy = -298.61571916 eV 16
13. Ir[Cor](PMe$_3$); C_s; S = 0; Total bonding energy = -306.08217303 eV 17
14. Ir[Cor](CO); C_s; S = 0; Total bonding energy = -257.47090108 eV 18
15. Ir[Cor](CO)(PMe$_3$); C_s; S = 0; Total bonding energy = -321.18162849 eV 19
16. Re[Cor](NMe); C_s; S = 0; Total bonding energy = -272.26987633 eV 20
17. Me$_3$PO; C_s; S = 0; Total bonding energy = -71.13474396 eV 21
18. CO; C_{lin}; S = 0; Total bonding energy = -14.68008335 eV . 21
19. PMe$_3$; C_s; S = 0; Total bonding energy = -63.38945444 eV . 22
20. MeN=NMe; C_{2h}; S = 0; Total bonding energy = -53.36675122 eV 22
21. Me$_3$P=NMe; C_1; S = 0; Total bonding energy = -90.40548752 eV 22

D. Optimized all-electron B3LYP/ZORA-STO-TZ2P Cartesian coordinates (Å) **23**

22. Ir[Cor](C); C_s; S = 0; Total bonding energy = -292.23355312 eV 23
23. Ir[Cor](C); C_s; S = 1; Total bonding energy = -290.45750677 eV 23

24. {Ir[Cor](C)}$^{-}$; C_s; S = ½; Total bonding energy = - -293.50312192 eV.. 24
25. Ir[Cor](NMe)$_2$; C_{2v}; S = 0; Total bonding energy = - -345.12164958 eV.. 25
26. Ir[Cor](NMe)$_2$; C_{2v}; S = 1; Total bonding energy = - -343.71958688 eV.. 26
27. {Ir[Cor](NMe)$_2$}$^{-}$; C_{2v}; S = ½; Total bonding energy = - -346.84301678 eV.. 27
28. Re[Cor](C); C_s; S = 0; Total bonding energy = -293.35782102 eV.. 28
29. Re[Cor](C); C_s; S = 1; Total bonding energy = -293.52018590 eV.. 29
30. {Re[Cor](C)}$^{-}$; C_s; S = ½; Total bonding energy = -296.62542075 eV.. 30
31. Re[Cor](NMe)$_2$; C_{2v}; S = 0; Total bonding energy = -346.20155412 eV.. 30
32. Re[Cor](NMe)$_2$; C_{2v}; S = 1; Total bonding energy = - -345.77665802 eV.. 31
33. {Re[Cor](NMe)$_2$}$^{-}$; C_{2v}; S = 1/2; Total bonding energy = -348.35796684 eV.. 32
34. Ir[Cor](PMe$_3$); C_s; S = 0; Total bonding energy = -359.51050008 eV.. 33
35. Ir[Cor](CO); C_s; S = 0; Total bonding energy = - -303.46545253 eV.. 34
36. Ir[Cor](CO)(PMe$_3$); C_s; S = 0; Total bonding energy = - -378.13715171 eV.. 35
37. Re[Cor](NMe); C_s; S = 0; Total bonding energy = -320.21619559 eV.. 36
38. Me$_3$PO; C_s; S = 0; Total bonding energy = -83.70709718 eV 37
39. CO; C_{lin}; S = 0; Total bonding energy = -18.10684518 eV . 38
40. PMe$_3$; C_s; S = 0; Total bonding energy = -73.88983796 eV. 38
41. MeN=NMe; C_{2h}; S = 0; Total bonding energy = -63.42932681 eV 38
42. Me$_3$P=NMe; C_1; S = 0; Total bonding energy = -105.95446898 eV 38

A. Computational method

All DFT calculations were carried out with the ADF 2018 program release r74086 2019-04-17,[1] the ZORA Hamiltonian, fine integration grids, and tight criteria for SCF cycles and geometry optimizations. All optimizations were done in the gas phase with scalar-relativistic DFT calculations with the OLYP[2,3] and B3LYP[4,5] functionals and all-electron ZORA[6,7]-STO-TZ2P basis sets. Additional details are provided in the sample input (p S5). Note that ADF does not provide conventional total energies, but rather total bonding energies, which are energies relative to those of the spherical, spin-restricted atoms. For calculating energy differences between two species, total bonding energies can be used exactly like total energies. See the ADF manual for further details.

---

(1) Velde, G. T.; Bickelhaupt, F. M.; Baerends, E. J.; Guerra, C. F.; van Gisbergen, S. J. A.; Snijders, J. G.; Ziegler, T. Chemistry with ADF. *J. Comput. Chem.* **2001**, *22*, 931-967.

(2) Handy, N. C.; Cohen, A. Left-Right Correlation Energy. *J. Mol. Phys*. **2001**, *99*, 403-412.

(3) T Lee, C.T.; Yang, W.T.; Parr, R.G. Development of the Colle-Salvetti Correlation-Energy Formula into a Functional of the Electron-Density. *Phys. Rev. B*, **1988**, *37*, 785-789.

(4) Becke, A. D. A New Mixing of Hartree–Fock and Local Density-Functional Theories. *J. Chem. Phys.* **1993**, *98*, 1372-1377.

(5) Stephens, P. J.; Devlin, F. J.; Chabalowski, C. F.; Frisch, M. J. Ab Initio Calculation of Vibrational Absorption and Circular Dichroism Spectra Using Density Functional Force Fields. *J. Phys. Chem.* **1994**, *98*, 11623-11627.

(6) van Lenthe, E.; Baerends, E. J.; Snijders, J. G. Relativistic Regular Two-Component Hamiltonians. *J. Chem. Phys*. **1993**, *99*, 4597-4610.

(7) van Lenthe, E.; Baerends, E. J.; Snijders, J. G. Relativistic Total Energy Using Regular Approximations. *J. Chem. Phys*. **1994**, *101*, 9783-9792.

B. Example input file

```
Title Ir[Cor](NMe)2, C2v, S = 0

COMMENT
END

UNITS
length angstrom
END

Atoms Cartesian
N        -0.017070000     -0.939209000      1.406852000
N         0.834731000      1.598057000     -1.217454000
N         0.834731000      1.598057000      1.217454000
N        -0.017070000     -0.939209000     -1.406852000
C        -0.388793000     -1.854338000      3.474157000
C        -0.721329000     -2.803735000      2.540089000
C         0.064674000     -0.685444000      2.765529000
C        -0.486793000     -2.225779000      1.242411000
C         0.482203000      0.525278000      3.339226000
C        -0.698099000     -2.830289000      0.000000000
C         0.844259000      1.644781000      2.590001000
C        -0.486793000     -2.225779000     -1.242411000
C         1.211783000      2.982936000      2.968578000
C        -0.721329000     -2.803735000     -2.540089000
C         1.427804000      3.706942000     -1.804407000
C         1.211783000      2.982936000     -2.968578000
C         1.184811000      2.818760000     -0.710901000
C         0.844259000      1.644781000     -2.590001000
C         0.482203000      0.525278000     -3.339226000
C         1.184811000      2.818760000      0.710901000
C         0.064674000     -0.685444000     -2.765529000
C         1.427804000      3.706942000      1.804407000
C        -0.388793000     -1.854338000     -3.474157000
C         2.578754000     -0.412985000      0.000000000
Ir        0.636248000      0.176948000      0.000000000
H         1.704283000      4.754379000     -1.726872000
H         1.277023000      3.349146000     -3.989348000
H         1.704283000      4.754379000      1.726872000
H         1.277023000      3.349146000      3.989348000
H         0.494102000      0.592562000      4.426656000
H        -0.458814000     -1.931719000      4.555866000
H        -1.105910000     -3.804921000      2.714044000
H        -1.074925000     -3.853000000      0.000000000
H        -1.105910000     -3.804921000     -2.714044000
H        -0.458814000     -1.931719000     -4.555866000
H         0.494102000      0.592562000     -4.426656000
end

SYMMETRY C(s)
```

```
CHARGE 0

INTEGRATION
accint 6.0
End

RELATIVISTIC Scalar ZORA

GEOMETRY
Iterations 500
Converge e=1.0E-3 grad=1.0E-3 rad=1.0E-2
End

SCF
Converge 1.0E-7
Iterations 500
END

BASIS
Type ZORA/TZ2P
Core None
END

XC
gga OLYP
END

endinput
```

C. Optimized all-electron OLYP/ZORA-STO-TZ2P Cartesian coordinates (Å).

1. Ir[Cor](C); C_s; S = 0; Total bonding energy = -248.34690085 eV

C	0.119235000	-0.708026000	2.785837000
C	0.119235000	-0.708026000	-2.785837000
C	0.516090000	0.517833000	3.336985000
C	0.516090000	0.517833000	-3.336985000
C	0.926942000	1.642006000	2.603246000
C	0.926942000	1.642006000	-2.603246000
C	1.143595000	3.009008000	2.962922000
C	1.143595000	3.009008000	-2.962922000
C	1.308347000	2.828301000	0.710347000
C	1.308347000	2.828301000	-0.710347000
C	1.369883000	3.735480000	1.799983000
C	1.369883000	3.735480000	-1.799983000
C	2.610247000	-0.577713000	0.000000000
C	-0.436982000	-2.274649000	1.251310000
C	-0.436982000	-2.274649000	-1.251310000
C	-0.446542000	-1.830529000	3.475237000
C	-0.446542000	-1.830529000	-3.475237000
C	-0.659828000	-2.861843000	0.000000000
C	-0.783779000	-2.782247000	2.545820000
C	-0.783779000	-2.782247000	-2.545820000
H	0.429440000	0.619520000	4.414136000
H	0.429440000	0.619520000	-4.414136000
H	1.098945000	3.402809000	3.970575000
H	1.098945000	3.402809000	-3.970575000
H	1.537632000	4.802227000	1.727347000
H	1.537632000	4.802227000	-1.727347000
H	-0.595446000	-1.880880000	4.546825000
H	-0.595446000	-1.880880000	-4.546825000
H	-1.103797000	-3.852291000	0.000000000
H	-1.251175000	-3.741709000	2.728649000
H	-1.251175000	-3.741709000	-2.728649000
Ir	1.034491000	0.040740000	0.000000000
N	0.121333000	-1.026971000	1.434857000
N	0.121333000	-1.026971000	-1.434857000
N	1.069881000	1.581412000	1.243301000
N	1.069881000	1.581412000	-1.243301000

2. Ir[Cor](C); C_s; S = 1; Total bonding energy = -246.54046220 eV

C	0.126072000	-0.707152000	2.787282000
C	0.126072000	-0.707152000	-2.787282000
C	0.550011000	0.492975000	3.344899000
C	0.550011000	0.492975000	-3.344899000

C	0.932388000	1.650423000	2.591980000
C	0.932388000	1.650423000	-2.591980000
C	1.128499000	3.022823000	2.958864000
C	1.128499000	3.022823000	-2.958864000
C	1.321721000	2.850126000	0.707889000
C	1.321721000	2.850126000	-0.707889000
C	1.362721000	3.754176000	1.795429000
C	1.362721000	3.754176000	-1.795429000
C	2.595717000	-0.584478000	0.000000000
C	-0.432179000	-1.850100000	3.481974000
C	-0.432179000	-1.850100000	-3.481974000
C	-0.474401000	-2.268286000	1.249040000
C	-0.474401000	-2.268286000	-1.249040000
C	-0.709349000	-2.852876000	0.000000000
C	-0.792024000	-2.784882000	2.553056000
C	-0.792024000	-2.784882000	-2.553056000
H	0.497646000	0.586447000	4.423849000
H	0.497646000	0.586447000	-4.423849000
H	1.067254000	3.412630000	3.966681000
H	1.067254000	3.412630000	-3.966681000
H	1.529877000	4.820945000	1.725290000
H	1.529877000	4.820945000	-1.725290000
H	-0.553237000	-1.910558000	4.556219000
H	-0.553237000	-1.910558000	-4.556219000
H	-1.151982000	-3.843823000	0.000000000
H	-1.255947000	-3.746138000	2.736555000
H	-1.255947000	-3.746138000	-2.736555000
Ir	1.015755000	0.038030000	0.000000000
N	0.092181000	-1.014683000	1.428282000
N	0.092181000	-1.014683000	-1.428282000
N	1.092302000	1.580861000	1.249885000
N	1.092302000	1.580861000	-1.249885000

3. {Ir[Cor](C)}$^{-}$; C_s; S = ½; Total bonding energy = -249.50413800 eV

C	0.117682000	-0.710317000	2.797417000
C	0.117682000	-0.710317000	-2.797417000
C	0.547707000	0.501774000	3.355296000
C	0.547707000	0.501774000	-3.355296000
C	0.924941000	1.656121000	2.618590000
C	0.924941000	1.656121000	-2.618590000
C	1.158519000	3.007755000	2.983521000
C	1.158519000	3.007755000	-2.983521000
C	1.280604000	2.858106000	0.719287000
C	1.280604000	2.858106000	-0.719287000
C	1.371822000	3.750611000	1.800638000
C	1.371822000	3.750611000	-1.800638000
C	2.565149000	-0.546135000	0.000000000
C	-0.424164000	-1.848559000	3.487465000

C	-0.424164000	-1.848559000	-3.487465000
C	-0.475659000	-2.276157000	1.254713000
C	-0.475659000	-2.276157000	-1.254713000
C	-0.702319000	-2.858548000	0.000000000
C	-0.783881000	-2.794994000	2.548589000
C	-0.783881000	-2.794994000	-2.548589000
H	0.501343000	0.585736000	4.436952000
H	0.501343000	0.585736000	-4.436952000
H	1.125614000	3.399817000	3.993322000
H	1.125614000	3.399817000	-3.993322000
H	1.551847000	4.816783000	1.732458000
H	1.551847000	4.816783000	-1.732458000
H	-0.545712000	-1.912673000	4.562641000
H	-0.545712000	-1.912673000	-4.562641000
H	-1.137037000	-3.854528000	0.000000000
H	-1.240978000	-3.760600000	2.733557000
H	-1.240978000	-3.760600000	-2.733557000
Ir	0.978682000	0.061573000	0.000000000
N	0.076573000	-1.012563000	1.439503000
N	0.076573000	-1.012563000	-1.439503000
N	1.029459000	1.606013000	1.250766000
N	1.029459000	1.606013000	-1.250766000

4. Ir[Cor](NMe)$_2$; C_{2v}; S = 0; Total bonding energy = -292.95974054 eV

C	0.000000000	0.000000000	-3.114442000
C	0.000000000	2.849371000	-0.859809000
C	0.000000000	-2.849371000	-0.859809000
C	0.719956000	0.000000000	3.010491000
C	1.273941000	0.000000000	-2.496768000
C	1.844469000	0.000000000	3.892067000
C	2.590010000	0.000000000	-3.091448000
C	2.613973000	0.000000000	1.726694000
C	2.803410000	0.000000000	-0.813644000
C	3.000724000	0.000000000	3.112574000
C	3.328246000	0.000000000	0.503999000
C	3.514765000	0.000000000	-2.070597000
C	-0.719956000	0.000000000	3.010491000
C	-1.273941000	0.000000000	-2.496768000
C	-1.844469000	0.000000000	3.892067000
C	-2.590010000	0.000000000	-3.091448000
C	-2.613973000	0.000000000	1.726694000
C	-2.803410000	0.000000000	-0.813644000
C	-3.000724000	0.000000000	3.112574000
C	-3.328246000	0.000000000	0.503999000
C	-3.514765000	0.000000000	-2.070597000
H	0.000000000	0.000000000	-4.200885000
H	0.000000000	3.811923000	-0.325751000
H	0.000000000	-3.811923000	-0.325751000

H	0.890709000	2.824070000	-1.508976000
H	0.890709000	-2.824070000	-1.508976000
H	1.805074000	0.000000000	4.974057000
H	2.795248000	0.000000000	-4.155162000
H	4.018817000	0.000000000	3.482187000
H	4.412348000	0.000000000	0.574813000
H	4.593296000	0.000000000	-2.173438000
H	-0.890709000	2.824070000	-1.508976000
H	-0.890709000	-2.824070000	-1.508976000
H	-1.805074000	0.000000000	4.974057000
H	-2.795248000	0.000000000	-4.155162000
H	-4.018817000	0.000000000	3.482187000
H	-4.412348000	0.000000000	0.574813000
H	-4.593296000	0.000000000	-2.173438000
Ir	0.000000000	0.000000000	0.245715000
N	0.000000000	1.806491000	0.079446000
N	0.000000000	-1.806491000	0.079446000
N	1.250579000	0.000000000	1.745928000
N	1.469582000	0.000000000	-1.143475000
N	-1.250579000	0.000000000	1.745928000
N	-1.469582000	0.000000000	-1.143475000

5. Ir[Cor](NMe)$_2$; *C*$_{2v}$; *S* = 1; Total bonding energy = -292.10392868 eV

C	0.000000000	0.000000000	-3.553664000
C	0.000000000	2.908355000	0.785392000
C	0.000000000	-2.908355000	0.785392000
C	0.719273000	0.000000000	2.558477000
C	1.267363000	0.000000000	-2.932757000
C	1.837021000	0.000000000	3.447065000
C	2.583732000	0.000000000	-3.528042000
C	2.610852000	0.000000000	1.280928000
C	2.797825000	0.000000000	-1.253578000
C	2.994194000	0.000000000	2.670620000
C	3.328938000	0.000000000	0.053862000
C	3.508495000	0.000000000	-2.510491000
C	-0.719273000	0.000000000	2.558477000
C	-1.267363000	0.000000000	-2.932757000
C	-1.837021000	0.000000000	3.447065000
C	-2.583732000	0.000000000	-3.528042000
C	-2.610852000	0.000000000	1.280928000
C	-2.797825000	0.000000000	-1.253578000
C	-2.994194000	0.000000000	2.670620000
C	-3.328938000	0.000000000	0.053862000
C	-3.508495000	0.000000000	-2.510491000
H	0.000000000	0.000000000	-4.639807000
H	0.000000000	2.539856000	1.819817000
H	0.000000000	-2.539856000	1.819817000
H	0.875226000	3.566189000	0.637753000

H	0.875226000	-3.566189000	0.637753000
H	1.791983000	0.000000000	4.528586000
H	2.786514000	0.000000000	-4.592087000
H	4.011426000	0.000000000	3.042831000
H	4.413202000	0.000000000	0.122294000
H	4.586859000	0.000000000	-2.612906000
H	-0.875226000	3.566189000	0.637753000
H	-0.875226000	-3.566189000	0.637753000
H	-1.791983000	0.000000000	4.528586000
H	-2.786514000	0.000000000	-4.592087000
H	-4.011426000	0.000000000	3.042831000
H	-4.413202000	0.000000000	0.122294000
H	-4.586859000	0.000000000	-2.612906000
Ir	0.000000000	0.000000000	-0.223974000
N	0.000000000	1.937230000	-0.234284000
N	0.000000000	-1.937230000	-0.234284000
N	1.254324000	0.000000000	1.286754000
N	1.454446000	0.000000000	-1.579857000
N	-1.254324000	0.000000000	1.286754000
N	-1.454446000	0.000000000	-1.579857000

6. {Ir[Cor](NMe)$_2$}$^-$; C_{2v}; S = ½; Total bonding energy = - -294.71565569 eV

C	0.000000000	0.000000000	-3.551777000
C	0.000000000	2.914803000	0.741947000
C	0.000000000	-2.914803000	0.741947000
C	0.724083000	0.000000000	2.573187000
C	1.270556000	0.000000000	-2.930550000
C	1.845436000	0.000000000	3.459730000
C	2.588948000	0.000000000	-3.528354000
C	2.607679000	0.000000000	1.293407000
C	2.792377000	0.000000000	-1.251429000
C	3.001732000	0.000000000	2.678272000
C	3.319509000	0.000000000	0.062116000
C	3.510877000	0.000000000	-2.507175000
C	-0.724083000	0.000000000	2.573187000
C	-1.270556000	0.000000000	-2.930550000
C	-1.845436000	0.000000000	3.459730000
C	-2.588948000	0.000000000	-3.528354000
C	-2.607679000	0.000000000	1.293407000
C	-2.792377000	0.000000000	-1.251429000
C	-3.001732000	0.000000000	2.678272000
C	-3.319509000	0.000000000	0.062116000
C	-3.510877000	0.000000000	-2.507175000
H	0.000000000	0.000000000	-4.638804000
H	0.000000000	2.576336000	1.791872000
H	0.000000000	-2.576336000	1.791872000
H	0.882293000	3.570437000	0.611887000
H	0.882293000	-3.570437000	0.611887000

H	1.804987000	0.000000000	4.542790000
H	2.794569000	0.000000000	-4.593084000
H	4.021233000	0.000000000	3.047061000
H	4.404946000	0.000000000	0.130187000
H	4.590825000	0.000000000	-2.606719000
H	-0.882293000	3.570437000	0.611887000
H	-0.882293000	-3.570437000	0.611887000
H	-1.804987000	0.000000000	4.542790000
H	-2.794569000	0.000000000	-4.593084000
H	-4.021233000	0.000000000	3.047061000
H	-4.404946000	0.000000000	0.130187000
H	-4.590825000	0.000000000	-2.606719000
Ir	0.000000000	0.000000000	-0.210307000
N	0.000000000	1.890387000	-0.235103000
N	0.000000000	-1.890387000	-0.235103000
N	1.249370000	0.000000000	1.310550000
N	1.458679000	0.000000000	-1.582629000
N	-1.249370000	0.000000000	1.310550000
N	-1.458679000	0.000000000	-1.582629000

7. Re[Cor](C); C_s; S = 0; Total bonding energy = -249.53386250 eV

C	0.365392000	-0.828265000	2.844286000
C	0.365392000	-0.828265000	-2.844286000
C	0.763326000	0.430222000	3.348574000
C	0.763326000	0.430222000	-3.348574000
C	1.024192000	1.585928000	2.592328000
C	1.024192000	1.585928000	-2.592328000
C	1.073159000	2.827301000	0.716320000
C	1.073159000	2.827301000	-0.716320000
C	1.149925000	2.965514000	2.972785000
C	1.149925000	2.965514000	-2.972785000
C	1.168961000	3.728971000	1.816688000
C	1.168961000	3.728971000	-1.816688000
C	2.849144000	-0.303859000	0.000000000
C	-0.364461000	-1.864854000	3.517295000
C	-0.364461000	-1.864854000	-3.517295000
C	-0.498165000	-2.209337000	1.271898000
C	-0.498165000	-2.209337000	-1.271898000
C	-0.820476000	-2.716523000	0.000000000
C	-0.901428000	-2.694034000	2.561572000
C	-0.901428000	-2.694034000	-2.561572000
H	0.705291000	0.552888000	4.426591000
H	0.705291000	0.552888000	-4.426591000
H	1.204427000	3.331585000	3.990675000
H	1.204427000	3.331585000	-3.990675000
H	1.236833000	4.807164000	1.753752000
H	1.236833000	4.807164000	-1.753752000
H	-0.517163000	-1.921843000	4.587922000

H	-0.517163000	-1.921843000	-4.587922000
H	-1.437229000	-3.610960000	0.000000000
H	-1.548229000	-3.546799000	2.725907000
H	-1.548229000	-3.546799000	-2.725907000
N	0.329228000	-1.113695000	1.496093000
N	0.329228000	-1.113695000	-1.496093000
N	1.004972000	1.564478000	1.218772000
N	1.004972000	1.564478000	-1.218772000
Re	1.151942000	-0.063044000	0.000000000

8. Re[Cor](C); C_s; S = 1; Total bonding energy = -249.70563716 eV

C	0.163717000	-0.736965000	2.801696000
C	0.163717000	-0.736965000	-2.801696000
C	0.552409000	0.518174000	3.331222000
C	0.552409000	0.518174000	-3.331222000
C	0.987267000	1.632179000	2.601879000
C	0.987267000	1.632179000	-2.601879000
C	1.103861000	3.019464000	2.958173000
C	1.103861000	3.019464000	-2.958173000
C	1.317553000	3.739553000	1.795797000
C	1.317553000	3.739553000	-1.795797000
C	1.353417000	2.801895000	0.719825000
C	1.353417000	2.801895000	-0.719825000
C	2.830385000	-0.647124000	0.000000000
C	-0.399080000	-2.284162000	1.270036000
C	-0.399080000	-2.284162000	-1.270036000
C	-0.485256000	-1.819028000	3.487382000
C	-0.485256000	-1.819028000	-3.487382000
C	-0.645019000	-2.842424000	0.000000000
C	-0.830075000	-2.764838000	2.552023000
C	-0.830075000	-2.764838000	-2.552023000
H	0.398119000	0.653578000	4.398119000
H	0.398119000	0.653578000	-4.398119000
H	0.991244000	3.422324000	3.956920000
H	0.991244000	3.422324000	-3.956920000
H	1.410948000	4.814358000	1.707351000
H	1.410948000	4.814358000	-1.707351000
H	-0.689979000	-1.847081000	4.550341000
H	-0.689979000	-1.847081000	-4.550341000
H	-1.163259000	-3.797496000	0.000000000
H	-1.360586000	-3.692996000	2.724121000
H	-1.360586000	-3.692996000	-2.724121000
N	0.231322000	-1.073195000	1.469117000
N	0.231322000	-1.073195000	-1.469117000
N	1.199336000	1.556580000	1.245504000
N	1.199336000	1.556580000	-1.245504000
Re	1.225365000	-0.056573000	0.000000000

9. {Re[Cor](C)}$^-$; C_s; S = ½; Total bonding energy = - -252.36158143 eV

C	0.190627000	-0.742531000	2.807454000
C	0.190627000	-0.742531000	-2.807454000
C	0.573144000	0.505643000	3.335605000
C	0.573144000	0.505643000	-3.335605000
C	1.021245000	1.627546000	2.601615000
C	1.021245000	1.627546000	-2.601615000
C	1.077442000	3.020289000	2.950756000
C	1.077442000	3.020289000	-2.950756000
C	1.299193000	3.742862000	1.782120000
C	1.299193000	3.742862000	-1.782120000
C	1.398960000	2.801257000	0.716457000
C	1.398960000	2.801257000	-0.716457000
C	2.886230000	-0.678913000	0.000000000
C	-0.374686000	-2.294177000	1.268393000
C	-0.374686000	-2.294177000	-1.268393000
C	-0.506428000	-1.805160000	3.486341000
C	-0.506428000	-1.805160000	-3.486341000
C	-0.624728000	-2.858213000	0.000000000
C	-0.852094000	-2.751712000	2.549767000
C	-0.852094000	-2.751712000	-2.549767000
H	0.386605000	0.652890000	4.396427000
H	0.386605000	0.652890000	-4.396427000
H	0.910786000	3.428236000	3.941235000
H	0.910786000	3.428236000	-3.941235000
H	1.347895000	4.821296000	1.687335000
H	1.347895000	4.821296000	-1.687335000
H	-0.746604000	-1.813544000	4.543636000
H	-0.746604000	-1.813544000	-4.543636000
H	-1.170114000	-3.798482000	0.000000000
H	-1.419766000	-3.660461000	2.715483000
H	-1.419766000	-3.660461000	-2.715483000
N	0.292642000	-1.109158000	1.478115000
N	0.292642000	-1.109158000	-1.478115000
N	1.285545000	1.547362000	1.259987000
N	1.285545000	1.547362000	-1.259987000
Re	1.275506000	-0.069607000	0.000000000

10. Re[Cor](NMe)$_2$; C_{2v}; S = 0; Total bonding energy = -296.38964416 eV

C	0.000000000	0.000000000	-2.979666000
C	0.000000000	2.968988000	-0.943205000
C	0.000000000	-2.968988000	-0.943205000
C	0.717322000	0.000000000	3.006962000
C	1.316180000	0.000000000	-2.411876000
C	1.828335000	0.000000000	3.893327000
C	2.613371000	0.000000000	1.729668000

C	2.619543000	0.000000000	-3.055032000
C	2.934263000	0.000000000	-0.785917000
C	2.984856000	0.000000000	3.116471000
C	3.402875000	0.000000000	0.549404000
C	3.592799000	0.000000000	-2.077180000
C	-0.717322000	0.000000000	3.006962000
C	-1.316180000	0.000000000	-2.411876000
C	-1.828335000	0.000000000	3.893327000
C	-2.613371000	0.000000000	1.729668000
C	-2.619543000	0.000000000	-3.055032000
C	-2.934263000	0.000000000	-0.785917000
C	-2.984856000	0.000000000	3.116471000
C	-3.402875000	0.000000000	0.549404000
C	-3.592799000	0.000000000	-2.077180000
H	0.000000000	0.000000000	-4.067178000
H	0.000000000	3.788245000	-0.206117000
H	0.000000000	-3.788245000	-0.206117000
H	0.895418000	3.095215000	-1.573177000
H	0.895418000	-3.095215000	-1.573177000
H	1.782145000	0.000000000	4.974931000
H	2.784058000	0.000000000	-4.126220000
H	4.003163000	0.000000000	3.486472000
H	4.479464000	0.000000000	0.692386000
H	4.664375000	0.000000000	-2.235775000
H	-0.895418000	3.095215000	-1.573177000
H	-0.895418000	-3.095215000	-1.573177000
H	-1.782145000	0.000000000	4.974931000
H	-2.784058000	0.000000000	-4.126220000
H	-4.003163000	0.000000000	3.486472000
H	-4.479464000	0.000000000	0.692386000
H	-4.664375000	0.000000000	-2.235775000
N	0.000000000	1.747389000	-0.260437000
N	0.000000000	-1.747389000	-0.260437000
N	1.232349000	0.000000000	1.733695000
N	1.608137000	0.000000000	-1.068065000
N	-1.232349000	0.000000000	1.733695000
N	-1.608137000	0.000000000	-1.068065000
Re	0.000000000	0.000000000	0.151383000

11. Re[Cor](NMe)$_2$; C_{2v}; S = 1; Total bonding energy = -295.84740414 eV (non Aufbau solution)

C	0.000000000	0.000000000	-3.049610000
C	0.000000000	2.952575000	-0.896992000
C	0.000000000	-2.952575000	-0.896992000
C	0.721116000	0.000000000	3.021532000
C	1.301032000	0.000000000	-2.460666000
C	1.838036000	0.000000000	3.903557000
C	2.612777000	0.000000000	-3.076395000
C	2.618732000	0.000000000	1.739917000
C	2.875954000	0.000000000	-0.801544000

C	2.996127000	0.000000000	3.125695000
C	3.366934000	0.000000000	0.529891000
C	3.560956000	0.000000000	-2.075624000
C	-0.721116000	0.000000000	3.021532000
C	-1.301032000	0.000000000	-2.460666000
C	-1.838036000	0.000000000	3.903557000
C	-2.612777000	0.000000000	-3.076395000
C	-2.618732000	0.000000000	1.739917000
C	-2.875954000	0.000000000	-0.801544000
C	-2.996127000	0.000000000	3.125695000
C	-3.366934000	0.000000000	0.529891000
C	-3.560956000	0.000000000	-2.075624000
H	0.000000000	0.000000000	-4.136539000
H	0.000000000	3.878108000	-0.302888000
H	0.000000000	-3.878108000	-0.302888000
H	0.882373000	2.958319000	-1.559704000
H	0.882373000	-2.958319000	-1.559704000
H	1.798018000	0.000000000	4.985682000
H	2.802552000	0.000000000	-4.143170000
H	4.012879000	0.000000000	3.499989000
H	4.448126000	0.000000000	0.636784000
H	4.636215000	0.000000000	-2.207980000
H	-0.882373000	2.958319000	-1.559704000
H	-0.882373000	-2.958319000	-1.559704000
H	-1.798018000	0.000000000	4.985682000
H	-2.802552000	0.000000000	-4.143170000
H	-4.012879000	0.000000000	3.499989000
H	-4.448126000	0.000000000	0.636784000
H	-4.636215000	0.000000000	-2.207980000
N	0.000000000	1.817861000	-0.085293000
N	0.000000000	-1.817861000	-0.085293000
N	1.247042000	0.000000000	1.750589000
N	1.545625000	0.000000000	-1.113065000
N	-1.247042000	0.000000000	1.750589000
N	-1.545625000	0.000000000	-1.113065000
Re	0.000000000	0.000000000	0.203743000

12. {Re[Cor](NMe)$_2$}$^-$; C_{2v}; S = 1/2; Total bonding energy = -298.61571916 eV

C	0.000000000	0.000000000	-3.090458000
C	0.000000000	2.993560000	-0.814233000
C	0.000000000	-2.993560000	-0.814233000
C	0.727184000	0.000000000	3.007897000
C	1.300833000	0.000000000	-2.496685000
C	1.847895000	0.000000000	3.892993000
C	2.612865000	0.000000000	1.724837000
C	2.618255000	0.000000000	-3.112489000
C	2.858212000	0.000000000	-0.833321000

C	3.005797000	0.000000000	3.110417000
C	3.350274000	0.000000000	0.501243000
C	3.559464000	0.000000000	-2.104256000
C	-0.727184000	0.000000000	3.007897000
C	-1.300833000	0.000000000	-2.496685000
C	-1.847895000	0.000000000	3.892993000
C	-2.612865000	0.000000000	1.724837000
C	-2.618255000	0.000000000	-3.112489000
C	-2.858212000	0.000000000	-0.833321000
C	-3.005797000	0.000000000	3.110417000
C	-3.350274000	0.000000000	0.501243000
C	-3.559464000	0.000000000	-2.104256000
H	0.000000000	0.000000000	-4.178513000
H	0.000000000	3.876960000	-0.149487000
H	0.000000000	-3.876960000	-0.149487000
H	0.889190000	3.089749000	-1.466934000
H	0.889190000	-3.089749000	-1.466934000
H	1.811219000	0.000000000	4.976478000
H	2.816672000	0.000000000	-4.178950000
H	4.025017000	0.000000000	3.481385000
H	4.433483000	0.000000000	0.600926000
H	4.636969000	0.000000000	-2.229426000
H	-0.889190000	3.089749000	-1.466934000
H	-0.889190000	-3.089749000	-1.466934000
H	-1.811219000	0.000000000	4.976478000
H	-2.816672000	0.000000000	-4.178950000
H	-4.025017000	0.000000000	3.481385000
H	-4.433483000	0.000000000	0.600926000
H	-4.636969000	0.000000000	-2.229426000
N	0.000000000	1.806091000	-0.069504000
N	0.000000000	-1.806091000	-0.069504000
N	1.249095000	0.000000000	1.746690000
N	1.533789000	0.000000000	-1.155173000
N	-1.249095000	0.000000000	1.746690000
N	-1.533789000	0.000000000	-1.155173000
Re	0.000000000	0.000000000	0.176841000

13. Ir[Cor](PMe$_3$); C_s; S = 0; Total bonding energy = -306.08217303 eV

C	0.335913000	-0.498299000	3.325034000
C	0.335913000	-0.498299000	-3.325034000
C	0.736254000	2.984281000	1.436875000
C	0.736254000	2.984281000	-1.436875000
C	1.545117000	-0.429567000	2.606540000
C	1.545117000	-0.429567000	-2.606540000
C	2.800505000	-0.385198000	0.717158000
C	2.800505000	-0.385198000	-0.717158000
C	2.918355000	-0.636175000	2.975881000
C	2.918355000	-0.636175000	-2.975881000

C	3.682600000	-0.613639000	1.817036000
C	3.682600000	-0.613639000	-1.817036000
C	-0.969119000	-0.499999000	2.789686000
C	-0.969119000	-0.499999000	-2.789686000
C	-1.746898000	2.854630000	0.000000000
C	-2.207300000	-0.732943000	3.487071000
C	-2.207300000	-0.732943000	-3.487071000
C	-2.638971000	-0.576632000	1.262338000
C	-2.638971000	-0.576632000	-1.262338000
C	-3.221056000	-0.780458000	2.563267000
C	-3.221056000	-0.780458000	-2.563267000
C	-3.256174000	-0.633140000	0.000000000
H	0.273847000	2.634015000	2.361572000
H	0.273847000	2.634015000	-2.361572000
H	0.407540000	-0.643003000	4.400132000
H	0.407540000	-0.643003000	-4.400132000
H	0.608884000	4.068078000	1.353109000
H	0.608884000	4.068078000	-1.353109000
H	1.800575000	2.744276000	1.465715000
H	1.800575000	2.744276000	-1.465715000
H	3.278702000	-0.819881000	3.980948000
H	3.278702000	-0.819881000	-3.980948000
H	4.751720000	-0.776039000	1.750487000
H	4.751720000	-0.776039000	-1.750487000
H	-1.694539000	3.948048000	0.000000000
H	-2.287305000	2.517872000	0.886302000
H	-2.287305000	2.517872000	-0.886302000
H	-2.293931000	-0.883600000	4.556967000
H	-2.293931000	-0.883600000	-4.556967000
H	-4.269711000	-0.976271000	2.753260000
H	-4.269711000	-0.976271000	-2.753260000
H	-4.329790000	-0.802494000	0.000000000
Ir	0.037435000	-0.018625000	0.000000000
N	1.540862000	-0.249002000	1.246459000
N	1.540862000	-0.249002000	-1.246459000
N	-1.284716000	-0.384007000	1.443403000
N	-1.284716000	-0.384007000	-1.443403000
P	-0.050909000	2.158097000	0.000000000

14. Ir[Cor](CO); C_s; S = 0; Total bonding energy = -257.47090108 eV

C	0.129664000	-0.724445000	2.788361000
C	0.129664000	-0.724445000	-2.788361000
C	0.521121000	0.515116000	3.329927000
C	0.521121000	0.515116000	-3.329927000
C	0.937186000	1.644871000	2.608934000
C	0.937186000	1.644871000	-2.608934000
C	1.155194000	3.014498000	2.973784000
C	1.155194000	3.014498000	-2.973784000

C	1.327569000	2.834272000	0.714125000
C	1.327569000	2.834272000	-0.714125000
C	1.389126000	3.739506000	1.814203000
C	1.389126000	3.739506000	-1.814203000
C	2.632667000	-0.551625000	0.000000000
C	-0.430320000	-2.296846000	1.258393000
C	-0.430320000	-2.296846000	-1.258393000
C	-0.441014000	-1.845730000	3.482351000
C	-0.441014000	-1.845730000	-3.482351000
C	-0.658297000	-2.873160000	0.000000000
C	-0.780774000	-2.799845000	2.556457000
C	-0.780774000	-2.799845000	-2.556457000
H	0.424115000	0.618902000	4.407265000
H	0.424115000	0.618902000	-4.407265000
H	1.105170000	3.410532000	3.980725000
H	1.105170000	3.410532000	-3.980725000
H	1.553539000	4.807973000	1.747355000
H	1.553539000	4.807973000	-1.747355000
H	-0.598419000	-1.891544000	4.553402000
H	-0.598419000	-1.891544000	-4.553402000
H	-1.114134000	-3.859542000	0.000000000
H	-1.258324000	-3.753697000	2.744868000
H	-1.258324000	-3.753697000	-2.744868000
Ir	0.951870000	0.068181000	0.000000000
N	0.130227000	-1.045126000	1.438516000
N	0.130227000	-1.045126000	-1.438516000
N	1.078384000	1.590632000	1.242148000
N	1.078384000	1.590632000	-1.242148000
O	3.727335000	-0.959422000	0.000000000

15. Ir[Cor](CO)(PMe_3); C_s; S = 0; Total bonding energy = -321.18162849 eV

C	0.631139000	2.234784000	0.000000000
C	0.930145000	-3.202486000	0.000000000
C	1.102478000	0.124457000	2.808437000
C	1.102478000	0.124457000	-2.808437000
C	2.328302000	-0.164097000	3.515379000
C	2.328302000	-0.164097000	-3.515379000
C	2.740479000	-0.261051000	1.274195000
C	2.740479000	-0.261051000	-1.274195000
C	3.319172000	-0.395832000	2.590997000
C	3.319172000	-0.395832000	-2.590997000
C	3.342969000	-0.392199000	0.000000000
C	-0.172294000	0.444133000	3.335109000
C	-0.172294000	0.444133000	-3.335109000
C	-1.363767000	0.730805000	2.617035000
C	-1.363767000	0.730805000	-2.617035000
C	-1.484284000	-2.620668000	1.434922000
C	-1.484284000	-2.620668000	-1.434922000

C	-2.605547000	1.001882000	0.722631000
C	-2.605547000	1.001882000	-0.722631000
C	-2.720599000	1.019843000	3.000178000
C	-2.720599000	1.019843000	-3.000178000
C	-3.476232000	1.182882000	1.842289000
C	-3.476232000	1.182882000	-1.842289000
H	0.566241000	-4.234638000	0.000000000
H	1.544692000	-3.036017000	0.885846000
H	1.544692000	-3.036017000	-0.885846000
H	2.430733000	-0.186331000	4.594138000
H	2.430733000	-0.186331000	-4.594138000
H	4.355951000	-0.635196000	2.796046000
H	4.355951000	-0.635196000	-2.796046000
H	4.404628000	-0.623353000	0.000000000
H	-0.239613000	0.469236000	4.419675000
H	-0.239613000	0.469236000	-4.419675000
H	-0.936944000	-2.437229000	2.360843000
H	-0.936944000	-2.437229000	-2.360843000
H	-1.686802000	-3.691974000	1.338868000
H	-1.686802000	-3.691974000	-1.338868000
H	-2.429303000	-2.076718000	1.478363000
H	-2.429303000	-2.076718000	-1.478363000
H	-3.087188000	1.093452000	4.017162000
H	-3.087188000	1.093452000	-4.017162000
H	-4.535105000	1.406942000	1.796432000
H	-4.535105000	1.406942000	-1.796432000
Ir	0.122740000	0.450693000	0.000000000
N	1.417251000	0.031562000	1.467449000
N	1.417251000	0.031562000	-1.467449000
N	-1.371721000	0.733973000	1.255170000
N	-1.371721000	0.733973000	-1.255170000
O	0.968521000	3.340524000	0.000000000
P	-0.494648000	-2.046751000	0.000000000

16. Re[Cor](NMe); C_s; S = 0; Total bonding energy = -272.26987633 eV

C	0.430418000	-0.018562000	3.336529000
C	0.430418000	-0.018562000	-3.336529000
C	1.613801000	0.072673000	2.594094000
C	1.613801000	0.072673000	-2.594094000
C	2.852357000	0.106195000	0.708044000
C	2.852357000	0.106195000	-0.708044000
C	2.986855000	-0.067261000	2.959002000
C	2.986855000	-0.067261000	-2.959002000
C	3.746029000	-0.056672000	1.794838000
C	3.746029000	-0.056672000	-1.794838000
C	-0.243041000	3.899059000	0.000000000
C	-0.852718000	-0.074221000	2.779893000
C	-0.852718000	-0.074221000	-2.779893000

C	-2.081737000	-0.319821000	3.469251000
C	-2.081737000	-0.319821000	-3.469251000
C	-2.499518000	-0.216269000	1.246845000
C	-2.499518000	-0.216269000	-1.246845000
C	-3.085661000	-0.408306000	2.536586000
C	-3.085661000	-0.408306000	-2.536586000
C	-3.126189000	-0.292929000	0.000000000
H	0.507714000	-0.126510000	4.414727000
H	0.507714000	-0.126510000	-4.414727000
H	0.736077000	4.397013000	0.000000000
H	3.356808000	-0.193356000	3.969607000
H	3.356808000	-0.193356000	-3.969607000
H	4.820136000	-0.178045000	1.724325000
H	4.820136000	-0.178045000	-1.724325000
H	-0.796960000	4.223123000	0.891390000
H	-0.796960000	4.223123000	-0.891390000
H	-2.172371000	-0.440094000	4.542568000
H	-2.172371000	-0.440094000	-4.542568000
H	-4.135850000	-0.603966000	2.718141000
H	-4.135850000	-0.603966000	-2.718141000
H	-4.195921000	-0.482077000	0.000000000
N	1.571997000	0.218515000	1.223600000
N	1.571997000	0.218515000	-1.223600000
N	-0.082243000	2.485901000	0.000000000
N	-1.138796000	0.018580000	1.414691000
N	-1.138796000	0.018580000	-1.414691000
Re	0.090811000	0.787543000	0.000000000

17. Me_3PO; C_s; S = 0; Total bonding energy = -71.13474396 eV

C	0.996838000	1.264877000	0.000000000
C	-1.181940000	0.027846000	1.444482000
C	-1.181940000	0.027846000	-1.444482000
H	0.418703000	2.194424000	0.000000000
H	1.635763000	1.238321000	0.887097000
H	1.635763000	1.238321000	-0.887097000
H	-0.585676000	-0.022707000	2.359911000
H	-0.585676000	-0.022707000	-2.359911000
H	-1.701601000	0.990599000	1.406709000
H	-1.701601000	0.990599000	-1.406709000
H	-1.920296000	-0.778681000	1.468769000
H	-1.920296000	-0.778681000	-1.468769000
O	0.654279000	-1.513229000	0.000000000
P	-0.084315000	-0.212721000	0.000000000

18. CO; C_{lin}; S = 0; Total bonding energy = -14.68008335 eV

C	0.000000000	0.000000000	-1.631366000
O	0.000000000	0.000000000	-2.768853000

19. PMe_3; C_s; S = 0; Total bonding energy = -63.38945444 eV

C	0.979098000	0.154063000	1.424873000
C	0.979098000	0.154063000	-1.424873000
C	-1.482165000	0.410540000	0.000000000
H	0.469559000	-0.108829000	2.357579000
H	0.469559000	-0.108829000	-2.357579000
H	1.054302000	1.245640000	1.355135000
H	1.054302000	1.245640000	-1.355135000
H	1.987616000	-0.269467000	1.470474000
H	1.987616000	-0.269467000	-1.470474000
H	-1.291838000	1.490231000	0.000000000
H	-2.076718000	0.153935000	0.882830000
H	-2.076718000	0.153935000	-0.882830000
P	0.067435000	-0.610512000	0.000000000

20. MeN=NMe; C_{2h}; S = 0; Total bonding energy = -53.36675122 eV

C	1.794402000	-0.210734000	0.000000000
C	-1.794402000	0.210734000	0.000000000
H	2.058407000	0.374267000	0.888044000
H	2.058407000	0.374267000	-0.888044000
H	2.346745000	-1.151249000	0.000000000
H	-2.058407000	-0.374267000	0.888044000
H	-2.058407000	-0.374267000	-0.888044000
H	-2.346745000	1.151249000	0.000000000
N	0.346393000	-0.515174000	0.000000000
N	-0.346393000	0.515174000	0.000000000

21. Me_3P=NMe; C_1; S = 0; Total bonding energy = -90.40548752 eV

C	1.011408000	1.352473000	-0.048828000
C	1.897228000	-2.104860000	0.405776000
C	-0.944176000	-0.144618000	1.424762000
C	-1.133608000	0.001980000	-1.472797000
H	0.357320000	2.220854000	0.078780000
H	1.575244000	-2.363743000	1.430788000
H	1.584143000	1.463448000	-0.974001000
H	1.714202000	1.318857000	0.789147000
H	2.254001000	-3.037627000	-0.049234000
H	2.782438000	-1.450924000	0.506742000
H	-0.282713000	-0.210335000	2.293910000
H	-0.586007000	0.062858000	-2.416428000
H	-1.517883000	0.785233000	1.491849000
H	-1.630546000	-0.995743000	1.448860000
H	-1.728455000	0.908538000	-1.332749000
H	-1.792136000	-0.869061000	-1.510306000

N	0.844432000	-1.551393000	-0.425148000
P	0.061577000	-0.230847000	-0.122847000

D. Optimized all-electron B3LYP/ZORA-STO-TZ2P Cartesian coordinates (Å).

22. Ir[Cor](C); C_s; S = 0; Total bonding energy = -292.23355312 eV

C	0.133354000	-0.709614000	2.783975000
C	0.133354000	-0.709614000	-2.783975000
C	0.526678000	0.513062000	3.332297000
C	0.526678000	0.513062000	-3.332297000
C	0.939986000	1.636164000	2.601878000
C	0.939986000	1.636164000	-2.601878000
C	1.134308000	3.005379000	2.958630000
C	1.134308000	3.005379000	-2.958630000
C	1.318669000	2.822720000	0.710057000
C	1.318669000	2.822720000	-0.710057000
C	1.357533000	3.730449000	1.798624000
C	1.357533000	3.730449000	-1.798624000
C	2.640447000	-0.593216000	0.000000000
C	-0.431077000	-2.271723000	1.248572000
C	-0.431077000	-2.271723000	-1.248572000
C	-0.448156000	-1.826318000	3.469690000
C	-0.448156000	-1.826318000	-3.469690000
C	-0.658997000	-2.855284000	0.000000000
C	-0.790512000	-2.771530000	2.542813000
C	-0.790512000	-2.771530000	-2.542813000
H	0.428127000	0.617965000	4.404416000
H	0.428127000	0.617965000	-4.404416000
H	1.072727000	3.401463000	3.959588000
H	1.072727000	3.401463000	-3.959588000
H	1.502443000	4.796138000	1.727378000
H	1.502443000	4.796138000	-1.727378000
H	-0.607759000	-1.871424000	4.535478000
H	-0.607759000	-1.871424000	-4.535478000
H	-1.118561000	-3.834331000	0.000000000
H	-1.272293000	-3.719277000	2.723540000
H	-1.272293000	-3.719277000	-2.723540000
Ir	1.071490000	0.026502000	0.000000000
N	0.139844000	-1.032785000	1.434551000
N	0.139844000	-1.032785000	-1.434551000
N	1.096891000	1.575526000	1.245349000
N	1.096891000	1.575526000	-1.245349000

23. Ir[Cor](C); C_s; S = 1; Total bonding energy = -290.45750677 eV

C	0.139137000	-0.703645000	2.781140000
C	0.139137000	-0.703645000	-2.781140000
C	0.559705000	0.488399000	3.340116000
C	0.559705000	0.488399000	-3.340116000
C	0.952846000	1.643588000	2.584933000
C	0.952846000	1.643588000	-2.584933000
C	1.106977000	3.019617000	2.947098000
C	1.106977000	3.019617000	-2.947098000
C	1.349623000	3.746464000	1.786582000
C	1.349623000	3.746464000	-1.786582000
C	1.355009000	2.836003000	0.705615000
C	1.355009000	2.836003000	-0.705615000
C	2.641308000	-0.625041000	0.000000000
C	-0.442546000	-1.840666000	3.473554000
C	-0.442546000	-1.840666000	-3.473554000
C	-0.460787000	-2.265004000	1.245194000
C	-0.460787000	-2.265004000	-1.245194000
C	-0.699407000	-2.846692000	0.000000000
C	-0.801376000	-2.770050000	2.546822000
C	-0.801376000	-2.770050000	-2.546822000
H	0.483979000	0.589982000	4.412980000
H	0.483979000	0.589982000	-4.412980000
H	1.007570000	3.413399000	3.945319000
H	1.007570000	3.413399000	-3.945319000
H	1.482232000	4.813316000	1.712531000
H	1.482232000	4.813316000	-1.712531000
H	-0.580250000	-1.892552000	4.541698000
H	-0.580250000	-1.892552000	-4.541698000
H	-1.159591000	-3.825515000	0.000000000
H	-1.283576000	-3.718147000	2.726242000
H	-1.283576000	-3.718147000	-2.726242000
Ir	1.078890000	0.014763000	0.000000000
N	0.119812000	-1.018238000	1.427463000
N	0.119812000	-1.018238000	-1.427463000
N	1.148999000	1.566810000	1.254165000
N	1.148999000	1.566810000	-1.254165000

24. {Ir[Cor](C)}$^-$; C_s; S = ½; Total bonding energy = - -293.50312192 eV

C	0.124716000	-0.707635000	2.792706000
C	0.124716000	-0.707635000	-2.792706000
C	0.547564000	0.499344000	3.350801000
C	0.547564000	0.499344000	-3.350801000
C	0.935628000	1.651812000	2.615437000
C	0.935628000	1.651812000	-2.615437000
C	1.143638000	3.000983000	2.976905000
C	1.143638000	3.000983000	-2.976905000
C	1.300678000	2.849999000	0.720802000
C	1.300678000	2.849999000	-0.720802000

C	1.363457000	3.741852000	1.794735000
C	1.363457000	3.741852000	-1.794735000
C	2.604495000	-0.569681000	0.000000000
C	-0.433212000	-1.840117000	3.476402000
C	-0.433212000	-1.840117000	-3.476402000
C	-0.461795000	-2.273436000	1.249907000
C	-0.461795000	-2.273436000	-1.249907000
C	-0.690603000	-2.852376000	0.000000000
C	-0.787532000	-2.782039000	2.540459000
C	-0.787532000	-2.782039000	-2.540459000
H	0.481227000	0.588660000	4.427214000
H	0.481227000	0.588660000	-4.427214000
H	1.088984000	3.395243000	3.979856000
H	1.088984000	3.395243000	-3.979856000
H	1.522400000	4.806717000	1.726806000
H	1.522400000	4.806717000	-1.726806000
H	-0.569906000	-1.898919000	4.545521000
H	-0.569906000	-1.898919000	-4.545521000
H	-1.136280000	-3.839431000	0.000000000
H	-1.256733000	-3.737435000	2.722374000
H	-1.256733000	-3.737435000	-2.722374000
Ir	1.031736000	0.044411000	0.000000000
N	0.099376000	-1.015260000	1.437604000
N	0.099376000	-1.015260000	-1.437604000
N	1.064788000	1.596803000	1.253060000
N	1.064788000	1.596803000	-1.253060000

25. Ir[Cor](NMe)$_2$; *C*$_{2v}$; *S* = 0; Total bonding energy = - -345.12164958 eV

C	0.000000000	0.000000000	-3.086513000
C	0.000000000	2.781972000	-0.939358000
C	0.000000000	-2.781972000	-0.939358000
C	0.719796000	0.000000000	3.026962000
C	1.272562000	0.000000000	-2.470494000
C	1.843298000	0.000000000	3.906402000
C	2.589826000	0.000000000	-3.062920000
C	2.611896000	0.000000000	1.746450000
C	2.801453000	0.000000000	-0.788802000
C	2.995897000	0.000000000	3.130927000
C	3.323675000	0.000000000	0.525377000
C	3.510121000	0.000000000	-2.046716000
C	-0.719796000	0.000000000	3.026962000
C	-1.272562000	0.000000000	-2.470494000
C	-1.843298000	0.000000000	3.906402000
C	-2.589826000	0.000000000	-3.062920000
C	-2.611896000	0.000000000	1.746450000
C	-2.801453000	0.000000000	-0.788802000
C	-2.995897000	0.000000000	3.130927000
C	-3.323675000	0.000000000	0.525377000

C	-3.510121000	0.000000000	-2.046716000
H	0.000000000	0.000000000	-4.168650000
H	0.000000000	3.772237000	-0.471811000
H	0.000000000	-3.772237000	-0.471811000
H	0.887863000	2.704768000	-1.582361000
H	0.887863000	-2.704768000	-1.582361000
H	1.804185000	0.000000000	4.983713000
H	2.794106000	0.000000000	-4.121922000
H	4.008790000	0.000000000	3.500990000
H	4.403811000	0.000000000	0.595721000
H	4.584032000	0.000000000	-2.149369000
H	-0.887863000	2.704768000	-1.582361000
H	-0.887863000	-2.704768000	-1.582361000
H	-1.804185000	0.000000000	4.983713000
H	-2.794106000	0.000000000	-4.121922000
H	-4.008790000	0.000000000	3.500990000
H	-4.403811000	0.000000000	0.595721000
H	-4.584032000	0.000000000	-2.149369000
Ir	0.000000000	0.000000000	0.265142000
N	0.000000000	1.798704000	0.069118000
N	0.000000000	-1.798704000	0.069118000
N	1.251806000	0.000000000	1.767449000
N	1.471037000	0.000000000	-1.122575000
N	-1.251806000	0.000000000	1.767449000
N	-1.471037000	0.000000000	-1.122575000

26. Ir[Cor](NMe)$_2$; C_{2v}; *S* = 1; Total bonding energy = - - 343.71958688 eV

C	0.000000000	0.000000000	-3.327887000
C	0.000000000	3.236879000	-0.016520000
C	0.000000000	-3.236879000	-0.016520000
C	0.737282000	0.000000000	2.748022000
C	1.279442000	0.000000000	-2.720671000
C	1.852398000	0.000000000	3.651046000
C	2.590515000	0.000000000	-3.322793000
C	2.617573000	0.000000000	1.495153000
C	2.790008000	0.000000000	-1.054675000
C	3.007043000	0.000000000	2.885567000
C	3.316373000	0.000000000	0.275720000
C	3.510983000	0.000000000	-2.305750000
C	-0.737282000	0.000000000	2.748022000
C	-1.279442000	0.000000000	-2.720671000
C	-1.852398000	0.000000000	3.651046000
C	-2.590515000	0.000000000	-3.322793000
C	-2.617573000	0.000000000	1.495153000
C	-2.790008000	0.000000000	-1.054675000
C	-3.007043000	0.000000000	2.885567000
C	-3.316373000	0.000000000	0.275720000
C	-3.510983000	0.000000000	-2.305750000

H	0.000000000	0.000000000	-4.411108000
H	0.000000000	3.617548000	1.018516000
H	0.000000000	-3.617548000	1.018516000
H	0.889679000	3.672529000	-0.503182000
H	0.889679000	-3.672529000	-0.503182000
H	1.800042000	0.000000000	4.727506000
H	2.792941000	0.000000000	-4.381939000
H	4.018968000	0.000000000	3.257805000
H	4.397789000	0.000000000	0.338635000
H	4.584951000	0.000000000	-2.404626000
H	-0.889679000	3.672529000	-0.503182000
H	-0.889679000	-3.672529000	-0.503182000
H	-1.800042000	0.000000000	4.727506000
H	-2.792941000	0.000000000	-4.381939000
H	-4.018968000	0.000000000	3.257805000
H	-4.397789000	0.000000000	0.338635000
H	-4.584951000	0.000000000	-2.404626000
Ir	0.000000000	0.000000000	-0.015613000
N	0.000000000	1.843492000	-0.023253000
N	0.000000000	-1.843492000	-0.023253000
N	1.258966000	0.000000000	1.511068000
N	1.470742000	0.000000000	-1.375735000
N	-1.258966000	0.000000000	1.511068000
N	-1.470742000	0.000000000	-1.375735000

27. {Ir[Cor](NMe)$_2$}$^-$; C_{2v}; S = ½; Total bonding energy = - -346.84301678 eV

C	0.000000000	0.000000000	-3.594955000
C	0.000000000	2.869441000	0.890178000
C	0.000000000	-2.869441000	0.890178000
C	0.729394000	0.000000000	2.513797000
C	1.266735000	0.000000000	-2.973202000
C	1.845846000	0.000000000	3.404925000
C	2.587355000	0.000000000	-3.567793000
C	2.610648000	0.000000000	1.246335000
C	2.787649000	0.000000000	-1.293208000
C	3.003939000	0.000000000	2.629128000
C	3.316140000	0.000000000	0.010982000
C	3.503969000	0.000000000	-2.550940000
C	-0.729394000	0.000000000	2.513797000
C	-1.266735000	0.000000000	-2.973202000
C	-1.845846000	0.000000000	3.404925000
C	-2.587355000	0.000000000	-3.567793000
C	-2.610648000	0.000000000	1.246335000
C	-2.787649000	0.000000000	-1.293208000
C	-3.003939000	0.000000000	2.629128000
C	-3.316140000	0.000000000	0.010982000
C	-3.503969000	0.000000000	-2.550940000
H	0.000000000	0.000000000	-4.678036000

H	0.000000000	2.429582000	1.901531000
H	0.000000000	-2.429582000	1.901531000
H	0.890130000	3.521929000	0.839453000
H	0.890130000	-3.521929000	0.839453000
H	1.800947000	0.000000000	4.482952000
H	2.792528000	0.000000000	-4.627711000
H	4.018026000	0.000000000	2.999124000
H	4.398161000	0.000000000	0.072533000
H	4.579372000	0.000000000	-2.649279000
H	-0.890130000	3.521929000	0.839453000
H	-0.890130000	-3.521929000	0.839453000
H	-1.800947000	0.000000000	4.482952000
H	-2.792528000	0.000000000	-4.627711000
H	-4.018026000	0.000000000	2.999124000
H	-4.398161000	0.000000000	0.072533000
H	-4.579372000	0.000000000	-2.649279000
Ir	0.000000000	0.000000000	-0.275361000
N	0.000000000	1.880904000	-0.110094000
N	0.000000000	-1.880904000	-0.110094000
N	1.259969000	0.000000000	1.263695000
N	1.453476000	0.000000000	-1.628274000
N	-1.259969000	0.000000000	1.263695000
N	-1.453476000	0.000000000	-1.628274000

28. Re[Cor](C); C_s; S = 0; Total bonding energy = -293.35782102 eV

C	0.328661000	0.605038000	3.297784000
C	0.328661000	0.605038000	-3.297784000
C	0.897583000	1.655756000	2.571333000
C	0.897583000	1.655756000	-2.571333000
C	1.070754000	3.042286000	2.900181000
C	1.070754000	3.042286000	-2.900181000
C	1.470325000	3.704655000	1.757959000
C	1.470325000	3.704655000	-1.757959000
C	1.562103000	2.732441000	0.719258000
C	1.562103000	2.732441000	-0.719258000
C	2.764849000	-0.846448000	0.000000000
C	-0.022641000	-0.653562000	2.783297000
C	-0.022641000	-0.653562000	-2.783297000
C	-0.241946000	-2.322371000	1.276869000
C	-0.241946000	-2.322371000	-1.276869000
C	-0.390145000	-2.899663000	0.000000000
C	-0.624166000	-1.776572000	3.443532000
C	-0.624166000	-1.776572000	-3.443532000
C	-0.741736000	-2.796242000	2.536162000
C	-0.741736000	-2.796242000	-2.536162000
H	0.104746000	0.783976000	4.341469000
H	0.104746000	0.783976000	-4.341469000
H	0.852940000	3.485240000	3.859055000

H	0.852940000	3.485240000	-3.859055000
H	1.632806000	4.764568000	1.650436000
H	1.632806000	4.764568000	-1.650436000
H	-0.812148000	-3.897506000	0.000000000
H	-0.935731000	-1.788743000	4.476017000
H	-0.935731000	-1.788743000	-4.476017000
H	-1.168228000	-3.772142000	2.705837000
H	-1.168228000	-3.772142000	-2.705837000
N	0.215039000	-1.034042000	1.481757000
N	0.215039000	-1.034042000	-1.481757000
N	1.272391000	1.517714000	1.256763000
N	1.272391000	1.517714000	-1.256763000
Re	1.227548000	-0.116318000	0.000000000

29. Re[Cor](C); C_s; S = 1; Total bonding energy = -293.52018590 eV

C	0.201078000	-0.734469000	2.797038000
C	0.201078000	-0.734469000	-2.797038000
C	0.563265000	0.499459000	3.319047000
C	0.563265000	0.499459000	-3.319047000
C	1.017636000	1.617688000	2.568005000
C	1.017636000	1.617688000	-2.568005000
C	1.034944000	3.028282000	2.905156000
C	1.034944000	3.028282000	-2.905156000
C	1.294863000	3.735205000	1.759714000
C	1.294863000	3.735205000	-1.759714000
C	1.452244000	2.783618000	0.699963000
C	1.452244000	2.783618000	-0.699963000
C	2.925536000	-0.743427000	0.000000000
C	-0.374508000	-2.271734000	1.256781000
C	-0.374508000	-2.271734000	-1.256781000
C	-0.502942000	-1.803995000	3.477838000
C	-0.502942000	-1.803995000	-3.477838000
C	-0.634255000	-2.840051000	0.000000000
C	-0.855736000	-2.729459000	2.550287000
C	-0.855736000	-2.729459000	-2.550287000
H	0.364177000	0.658494000	4.370340000
H	0.364177000	0.658494000	-4.370340000
H	0.831403000	3.435689000	3.882705000
H	0.831403000	3.435689000	-3.882705000
H	1.333468000	4.807230000	1.656288000
H	1.333468000	4.807230000	-1.656288000
H	-0.735639000	-1.811251000	4.530774000
H	-0.735639000	-1.811251000	-4.530774000
H	-1.188642000	-3.768575000	0.000000000
H	-1.421969000	-3.633312000	2.710487000
H	-1.421969000	-3.633312000	-2.710487000
N	0.302085000	-1.107725000	1.461929000
N	0.302085000	-1.107725000	-1.461929000

N	1.348878000	1.516789000	1.268669000
N	1.348878000	1.516789000	-1.268669000
Re	1.326775000	-0.092897000	0.000000000

30. {Re[Cor](C)}$^-$; C_s; S = ½; Total bonding energy = -296.62542075 eV

C	0.196318000	-0.736410000	2.797861000
C	0.196318000	-0.736410000	-2.797861000
C	0.570480000	0.505561000	3.326551000
C	0.570480000	0.505561000	-3.326551000
C	1.030757000	1.621589000	2.597306000
C	1.030757000	1.621589000	-2.597306000
C	1.057591000	3.014335000	2.935902000
C	1.057591000	3.014335000	-2.935902000
C	1.288977000	3.729020000	1.770555000
C	1.288977000	3.729020000	-1.770555000
C	1.424486000	2.784123000	0.715650000
C	1.424486000	2.784123000	-0.715650000
C	2.935632000	-0.720939000	0.000000000
C	-0.362904000	-2.285919000	1.261984000
C	-0.362904000	-2.285919000	-1.261984000
C	-0.514724000	-1.791830000	3.471571000
C	-0.514724000	-1.791830000	-3.471571000
C	-0.616398000	-2.850070000	0.000000000
C	-0.854402000	-2.735011000	2.540468000
C	-0.854402000	-2.735011000	-2.540468000
H	0.364175000	0.656857000	4.379230000
H	0.364175000	0.656857000	-4.379230000
H	0.864262000	3.424966000	3.915388000
H	0.864262000	3.424966000	-3.915388000
H	1.318216000	4.802767000	1.668931000
H	1.318216000	4.802767000	-1.668931000
H	-0.764557000	-1.792698000	4.521918000
H	-0.764557000	-1.792698000	-4.521918000
H	-1.169143000	-3.781298000	0.000000000
H	-1.427864000	-3.635394000	2.700985000
H	-1.427864000	-3.635394000	-2.700985000
N	0.307999000	-1.110082000	1.472738000
N	0.307999000	-1.110082000	-1.472738000
N	1.323378000	1.536861000	1.266855000
N	1.323378000	1.536861000	-1.266855000
Re	1.341441000	-0.089098000	0.000000000

31. Re[Cor](NMe)$_2$; C_{2v}; S = 0; Total bonding energy = -346.20155412 eV

C	0.000000000	0.000000000	-2.917868000
C	0.000000000	1.743534000	-0.335068000

C	0.000000000	2.826838000	-1.280716000
C	0.000000000	-1.743534000	-0.335068000
C	0.000000000	-2.826838000	-1.280716000
C	0.723900000	0.000000000	3.122192000
C	1.318545000	0.000000000	-2.344367000
C	1.841980000	0.000000000	4.004966000
C	2.621525000	0.000000000	1.845367000
C	2.633312000	0.000000000	-2.970165000
C	2.916780000	0.000000000	-0.693293000
C	2.997465000	0.000000000	3.231589000
C	3.384174000	0.000000000	0.643068000
C	3.588442000	0.000000000	-1.981811000
C	-0.723900000	0.000000000	3.122192000
C	-1.318545000	0.000000000	-2.344367000
C	-1.841980000	0.000000000	4.004966000
C	-2.621525000	0.000000000	1.845367000
C	-2.633312000	0.000000000	-2.970165000
C	-2.916780000	0.000000000	-0.693293000
C	-2.997465000	0.000000000	3.231589000
C	-3.384174000	0.000000000	0.643068000
C	-3.588442000	0.000000000	-1.981811000
H	0.000000000	0.000000000	-4.001592000
H	0.000000000	3.806839000	-0.796566000
H	0.000000000	-3.806839000	-0.796566000
H	0.878458000	2.736922000	-1.933529000
H	0.878458000	-2.736922000	-1.933529000
H	1.801225000	0.000000000	5.082351000
H	2.816646000	0.000000000	-4.033553000
H	4.009308000	0.000000000	3.605490000
H	4.459610000	0.000000000	0.768828000
H	4.657446000	0.000000000	-2.127313000
H	-0.878458000	2.736922000	-1.933529000
H	-0.878458000	-2.736922000	-1.933529000
H	-1.801225000	0.000000000	5.082351000
H	-2.816646000	0.000000000	-4.033553000
H	-4.009308000	0.000000000	3.605490000
H	-4.459610000	0.000000000	0.768828000
H	-4.657446000	0.000000000	-2.127313000
N	1.249591000	0.000000000	1.859648000
N	1.594316000	0.000000000	-1.004684000
N	-1.249591000	0.000000000	1.859648000
N	-1.594316000	0.000000000	-1.004684000
Re	0.000000000	0.000000000	0.300464000

32. Re[Cor](NMe)$_2$; C_{2v}; S = 1; Total bonding energy = - -345.77665802 eV

C	0.000000000	0.000000000	-2.976886000
C	0.000000000	1.821622000	-0.264546000
C	0.000000000	3.017209000	-1.094625000

C	0.000000000	-1.821622000	-0.264546000
C	0.000000000	-3.017209000	-1.094625000
C	0.735412000	0.000000000	3.061486000
C	1.319875000	0.000000000	-2.406932000
C	1.858893000	0.000000000	3.957623000
C	2.621906000	0.000000000	1.795594000
C	2.630240000	0.000000000	-3.036552000
C	2.880609000	0.000000000	-0.764727000
C	3.008709000	0.000000000	3.189007000
C	3.356975000	0.000000000	0.593296000
C	3.576965000	0.000000000	-2.039846000
C	-0.735412000	0.000000000	3.061486000
C	-1.319875000	0.000000000	-2.406932000
C	-1.858893000	0.000000000	3.957623000
C	-2.621906000	0.000000000	1.795594000
C	-2.630240000	0.000000000	-3.036552000
C	-2.880609000	0.000000000	-0.764727000
C	-3.008709000	0.000000000	3.189007000
C	-3.356975000	0.000000000	0.593296000
C	-3.576965000	0.000000000	-2.039846000
H	0.000000000	0.000000000	-4.061674000
H	0.000000000	3.915609000	-0.466332000
H	0.000000000	-3.915609000	-0.466332000
H	0.887532000	3.065437000	-1.739989000
H	0.887532000	-3.065437000	-1.739989000
H	1.814396000	0.000000000	5.034705000
H	2.819309000	0.000000000	-4.098611000
H	4.021138000	0.000000000	3.560773000
H	4.435027000	0.000000000	0.703611000
H	4.647367000	0.000000000	-2.172836000
H	-0.887532000	3.065437000	-1.739989000
H	-0.887532000	-3.065437000	-1.739989000
H	-1.814396000	0.000000000	5.034705000
H	-2.819309000	0.000000000	-4.098611000
H	-4.021138000	0.000000000	3.560773000
H	-4.435027000	0.000000000	0.703611000
H	-4.647367000	0.000000000	-2.172836000
N	1.246313000	0.000000000	1.815444000
N	1.570741000	0.000000000	-1.068216000
N	-1.246313000	0.000000000	1.815444000
N	-1.570741000	0.000000000	-1.068216000
Re	0.000000000	0.000000000	0.240701000

33. $\{Re[Cor](NMe)_2\}^-$; C_{2v}; S = 1/2; Total bonding energy = -348.35796684 eV

C	0.000000000	0.000000000	-2.963727000
C	0.000000000	1.809628000	-0.264443000
C	0.000000000	2.942537000	-1.184081000
C	0.000000000	-1.809628000	-0.264443000

C	0.000000000	-2.942537000	-1.184081000
C	0.728055000	0.000000000	3.101084000
C	1.312046000	0.000000000	-2.380563000
C	1.847792000	0.000000000	3.986240000
C	2.617431000	0.000000000	1.823062000
C	2.631305000	0.000000000	-3.006604000
C	2.888259000	0.000000000	-0.728372000
C	3.005322000	0.000000000	3.208900000
C	3.365584000	0.000000000	0.605932000
C	3.578447000	0.000000000	-2.011760000
C	-0.728055000	0.000000000	3.101084000
C	-1.312046000	0.000000000	-2.380563000
C	-1.847792000	0.000000000	3.986240000
C	-2.617431000	0.000000000	1.823062000
C	-2.631305000	0.000000000	-3.006604000
C	-2.888259000	0.000000000	-0.728372000
C	-3.005322000	0.000000000	3.208900000
C	-3.365584000	0.000000000	0.605932000
C	-3.578447000	0.000000000	-2.011760000
H	0.000000000	0.000000000	-4.048324000
H	0.000000000	3.890257000	-0.631925000
H	0.000000000	-3.890257000	-0.631925000
H	0.885385000	2.934037000	-1.835733000
H	0.885385000	-2.934037000	-1.835733000
H	1.809350000	0.000000000	5.064855000
H	2.821600000	0.000000000	-4.069870000
H	4.018961000	0.000000000	3.581085000
H	4.443800000	0.000000000	0.718816000
H	4.649802000	0.000000000	-2.148372000
H	-0.885385000	2.934037000	-1.835733000
H	-0.885385000	-2.934037000	-1.835733000
H	-1.809350000	0.000000000	5.064855000
H	-2.821600000	0.000000000	-4.069870000
H	-4.018961000	0.000000000	3.581085000
H	-4.443800000	0.000000000	0.718816000
H	-4.649802000	0.000000000	-2.148372000
N	1.252393000	0.000000000	1.843397000
N	1.568050000	0.000000000	-1.045694000
N	-1.252393000	0.000000000	1.843397000
N	-1.568050000	0.000000000	-1.045694000
Re	0.000000000	0.000000000	0.270420000

34. Ir[Cor](PMe_3); C_s; S = 0; Total bonding energy = -359.51050008 eV

C	0.331003000	-0.485920000	3.319268000
C	0.331003000	-0.485920000	-3.319268000
C	0.745746000	2.925627000	1.440123000
C	0.745746000	2.925627000	-1.440123000
C	1.543899000	-0.419116000	2.606259000

C	1.543899000	-0.419116000	-2.606259000
C	2.802681000	-0.370478000	0.718932000
C	2.802681000	-0.370478000	-0.718932000
C	2.919254000	-0.603506000	2.978910000
C	2.919254000	-0.603506000	-2.978910000
C	3.684090000	-0.578887000	1.823420000
C	3.684090000	-0.578887000	-1.823420000
C	-0.972860000	-0.493105000	2.788921000
C	-0.972860000	-0.493105000	-2.788921000
C	-1.744891000	2.812360000	0.000000000
C	-2.215607000	-0.711022000	3.487159000
C	-2.215607000	-0.711022000	-3.487159000
C	-2.643656000	-0.565302000	1.261942000
C	-2.643656000	-0.565302000	-1.261942000
C	-3.226560000	-0.754488000	2.567064000
C	-3.226560000	-0.754488000	-2.567064000
C	-3.259306000	-0.613066000	0.000000000
H	0.280889000	2.562793000	2.355772000
H	0.280889000	2.562793000	-2.355772000
H	0.400664000	-0.618064000	4.391957000
H	0.400664000	-0.618064000	-4.391957000
H	0.639562000	4.009968000	1.379714000
H	0.639562000	4.009968000	-1.379714000
H	1.801586000	2.661263000	1.461346000
H	1.801586000	2.661263000	-1.461346000
H	3.281580000	-0.770190000	3.980991000
H	3.281580000	-0.770190000	-3.980991000
H	4.751052000	-0.723599000	1.763616000
H	4.751052000	-0.723599000	-1.763616000
H	-1.710724000	3.903270000	0.000000000
H	-2.277983000	2.464292000	0.883253000
H	-2.277983000	2.464292000	-0.883253000
H	-2.304908000	-0.848160000	4.553754000
H	-2.304908000	-0.848160000	-4.553754000
H	-4.273100000	-0.931921000	2.758558000
H	-4.273100000	-0.931921000	-2.758558000
H	-4.330947000	-0.767627000	0.000000000
Ir	0.033820000	-0.051007000	0.000000000
N	1.547017000	-0.253409000	1.250018000
N	1.547017000	-0.253409000	-1.250018000
N	-1.294600000	-0.390066000	1.447748000
N	-1.294600000	-0.390066000	-1.447748000
P	-0.050790000	2.136786000	0.000000000

35. Ir[Cor](CO); C_s; S = 0; Total bonding energy = - -303.46545253 eV

C	0.134358000	-0.723388000	2.784710000
C	0.134358000	-0.723388000	-2.784710000
C	0.522062000	0.515121000	3.322387000

C	0.522062000	0.515121000	-3.322387000
C	0.943019000	1.642728000	2.606008000
C	0.943019000	1.642728000	-2.606008000
C	1.145573000	3.014838000	2.968847000
C	1.145573000	3.014838000	-2.968847000
C	1.341118000	2.829202000	0.714048000
C	1.341118000	2.829202000	-0.714048000
C	1.381365000	3.736868000	1.812850000
C	1.381365000	3.736868000	-1.812850000
C	2.658250000	-0.570935000	0.000000000
C	-0.424816000	-2.294987000	1.256337000
C	-0.424816000	-2.294987000	-1.256337000
C	-0.445914000	-1.841140000	3.476887000
C	-0.445914000	-1.841140000	-3.476887000
C	-0.653002000	-2.868517000	0.000000000
C	-0.784076000	-2.792562000	2.554524000
C	-0.784076000	-2.792562000	-2.554524000
H	0.413100000	0.621955000	4.394051000
H	0.413100000	0.621955000	-4.394051000
H	1.082700000	3.411805000	3.969335000
H	1.082700000	3.411805000	-3.969335000
H	1.534327000	4.801988000	1.746294000
H	1.534327000	4.801988000	-1.746294000
H	-0.607397000	-1.884183000	4.542556000
H	-0.607397000	-1.884183000	-4.542556000
H	-1.115032000	-3.847268000	0.000000000
H	-1.263967000	-3.740001000	2.741795000
H	-1.263967000	-3.740001000	-2.741795000
Ir	0.970104000	0.059519000	0.000000000
N	0.140448000	-1.051175000	1.440427000
N	0.140448000	-1.051175000	-1.440427000
N	1.103393000	1.589576000	1.245655000
N	1.103393000	1.589576000	-1.245655000
O	3.733421000	-0.983520000	0.000000000

36. Ir[Cor](CO)(PMe_3); C_s; S = 0; Total bonding energy = - -378.13715171 eV

C	0.657593000	2.267895000	0.000000000
C	0.942562000	-3.095068000	0.000000000
C	1.099307000	0.091519000	2.806237000
C	1.099307000	0.091519000	-2.806237000
C	2.323048000	-0.214149000	3.513183000
C	2.323048000	-0.214149000	-3.513183000
C	2.736732000	-0.288264000	1.273133000
C	2.736732000	-0.288264000	-1.273133000
C	3.311215000	-0.441202000	2.592213000
C	3.311215000	-0.441202000	-2.592213000
C	3.337853000	-0.421022000	0.000000000
C	-0.174776000	0.401398000	3.328589000

C	-0.174776000	0.401398000	-3.328589000
C	-1.367512000	0.697301000	2.614642000
C	-1.367512000	0.697301000	-2.614642000
C	-1.474264000	-2.492150000	1.439502000
C	-1.474264000	-2.492150000	-1.439502000
C	-2.607966000	0.974852000	0.724162000
C	-2.607966000	0.974852000	-0.724162000
C	-2.731251000	0.943471000	2.998994000
C	-2.731251000	0.943471000	-2.998994000
C	-3.485776000	1.109128000	1.843840000
C	-3.485776000	1.109128000	-1.843840000
H	0.593112000	-4.128529000	0.000000000
H	1.554260000	-2.918462000	0.882925000
H	1.554260000	-2.918462000	-0.882925000
H	2.422507000	-0.248126000	4.587063000
H	2.422507000	-0.248126000	-4.587063000
H	4.341078000	-0.688599000	2.796429000
H	4.341078000	-0.688599000	-2.796429000
H	4.392791000	-0.662575000	0.000000000
H	-0.246599000	0.402649000	4.408979000
H	-0.246599000	0.402649000	-4.408979000
H	-0.921117000	-2.298063000	2.357074000
H	-0.921117000	-2.298063000	-2.357074000
H	-1.694071000	-3.558075000	1.364490000
H	-1.694071000	-3.558075000	-1.364490000
H	-2.406403000	-1.930159000	1.476527000
H	-2.406403000	-1.930159000	-1.476527000
H	-3.101969000	0.988209000	4.010866000
H	-3.101969000	0.988209000	-4.010866000
H	-4.544895000	1.306292000	1.800572000
H	-4.544895000	1.306292000	-1.800572000
Ir	0.125932000	0.441136000	0.000000000
N	1.424294000	0.018778000	1.469826000
N	1.424294000	0.018778000	-1.469826000
N	-1.372823000	0.741548000	1.258775000
N	-1.372823000	0.741548000	-1.258775000
O	1.000044000	3.353168000	0.000000000
P	-0.484867000	-1.956115000	0.000000000

37. Re[Cor](NMe); C_s; S = 0; Total bonding energy = -320.21619559 eV

C	0.433103000	-0.014594000	3.328243000
C	0.433103000	-0.014594000	-3.328243000
C	1.612914000	0.087585000	2.589382000
C	1.612914000	0.087585000	-2.589382000
C	2.843907000	0.133599000	0.706160000
C	2.843907000	0.133599000	-0.706160000
C	2.977511000	-0.118909000	2.944910000
C	2.977511000	-0.118909000	-2.944910000

C	3.731695000	-0.101256000	1.782216000
C	3.731695000	-0.101256000	-1.782216000
C	-0.270110000	3.995997000	0.000000000
C	-0.844570000	-0.052905000	2.772477000
C	-0.844570000	-0.052905000	-2.772477000
C	-2.066796000	-0.341126000	3.455866000
C	-2.066796000	-0.341126000	-3.455866000
C	-2.486715000	-0.194006000	1.241318000
C	-2.486715000	-0.194006000	-1.241318000
C	-3.065612000	-0.430483000	2.526067000
C	-3.065612000	-0.430483000	-2.526067000
C	-3.112078000	-0.279016000	0.000000000
H	0.510181000	-0.163782000	4.397154000
H	0.510181000	-0.163782000	-4.397154000
H	0.698341000	4.502489000	0.000000000
H	3.341398000	-0.299142000	3.944129000
H	3.341398000	-0.299142000	-3.944129000
H	4.793909000	-0.268141000	1.703678000
H	4.793909000	-0.268141000	-1.703678000
H	-0.826053000	4.306911000	0.888020000
H	-0.826053000	4.306911000	-0.888020000
H	-2.150323000	-0.495578000	4.520387000
H	-2.150323000	-0.495578000	-4.520387000
H	-4.104445000	-0.662035000	2.700797000
H	-4.104445000	-0.662035000	-2.700797000
H	-4.171541000	-0.498851000	0.000000000
N	1.575427000	0.285059000	1.229166000
N	1.575427000	0.285059000	-1.229166000
N	-0.087673000	2.582869000	0.000000000
N	-1.136225000	0.069316000	1.413807000
N	-1.136225000	0.069316000	-1.413807000
Re	0.088947000	0.882000000	0.000000000

38. Me_3PO; C_s; S = 0; Total bonding energy = -83.70709718 eV

C	0.995842000	1.264531000	0.000000000
C	-1.182218000	0.029278000	1.444386000
C	-1.182218000	0.029278000	-1.444386000
H	0.432962000	2.198673000	0.000000000
H	1.632953000	1.226175000	0.883779000
H	1.632953000	1.226175000	-0.883779000
H	-0.579461000	-0.031299000	2.350846000
H	-0.579461000	-0.031299000	-2.350846000
H	-1.704515000	0.986435000	1.421408000
H	-1.704515000	0.986435000	-1.421408000
H	-1.911826000	-0.780710000	1.463699000
H	-1.911826000	-0.780710000	-1.463699000
O	0.635838000	-1.485512000	0.000000000
P	-0.096500000	-0.193347000	0.000000000

39. CO; *C*$_{lin}$; *S* = 0; Total bonding energy = -18.10684518 eV

C	0.000000000	0.000000000	-1.637338000
O	0.000000000	0.000000000	-2.762881000

40. PMe_3; *C*$_s$; *S* = 0; Total bonding energy = -73.88983796 eV

C	0.972749000	0.148507000	1.415358000
C	0.972749000	0.148507000	-1.415358000
C	-1.471666000	0.406311000	0.000000000
H	0.471479000	-0.100480000	2.351693000
H	0.471479000	-0.100480000	-2.351693000
H	1.031035000	1.236039000	1.322991000
H	1.031035000	1.236039000	-1.322991000
H	1.984721000	-0.256067000	1.462973000
H	1.984721000	-0.256067000	-1.462973000
H	-1.252313000	1.476966000	0.000000000
H	-2.069315000	0.167932000	0.880980000
H	-2.069315000	0.167932000	-0.880980000
P	0.063786000	-0.634194000	0.000000000

41. MeN=NMe; *C*$_{2h}$; *S* = 0; Total bonding energy = -63.42932681 eV

C	1.798822000	-0.063912000	0.000000000
C	-1.798822000	0.063912000	0.000000000
H	1.833834000	-1.155606000	0.000000000
H	2.305681000	0.339184000	0.879000000
H	2.305681000	0.339184000	-0.879000000
H	-1.833834000	1.155606000	0.000000000
H	-2.305681000	-0.339184000	0.879000000
H	-2.305681000	-0.339184000	-0.879000000
N	0.426973000	0.444461000	0.000000000
N	-0.426973000	-0.444461000	0.000000000

42. Me_3P=NMe; *C*$_1$; *S* = 0; Total bonding energy = -105.95446898 eV

C	1.007308000	1.343661000	-0.056189000
C	1.901978000	-2.095207000	0.411974000
C	-0.937133000	-0.155193000	1.417840000
C	-1.139551000	0.007391000	-1.466213000
H	0.358511000	2.206933000	0.099113000
H	1.556419000	1.467048000	-0.989841000
H	1.578395000	-2.353892000	1.431104000
H	1.727740000	1.295450000	0.761544000
H	2.256446000	-3.022003000	-0.045125000
H	2.780714000	-1.440340000	0.510719000
H	-0.274620000	-0.253683000	2.278867000
H	-0.601224000	0.054544000	-2.411825000
H	-1.488548000	0.782097000	1.505350000

H	-1.638416000	-0.989721000	1.427028000
H	-1.715640000	0.922338000	-1.325604000
H	-1.812179000	-0.848757000	-1.493752000
N	0.849109000	-1.535510000	-0.409519000
P	0.057159000	-0.230065000	-0.127195000
